# An Efficient Multistrategy DNA Decontamination Procedure of PCR Reagents for Hypersensitive PCR Applications

**DOI:** 10.1371/journal.pone.0013042

**Published:** 2010-09-28

**Authors:** Sophie Champlot, Camille Berthelot, Mélanie Pruvost, E. Andrew Bennett, Thierry Grange, Eva-Maria Geigl

**Affiliations:** Institut Jacques Monod, UMR7592 CNRS, Université Paris 7, Paris, France; Institute of Evolutionary Biology (CSIC-UPF), Spain

## Abstract

**Background:**

PCR amplification of minute quantities of degraded DNA for ancient DNA research, forensic analyses, wildlife studies and ultrasensitive diagnostics is often hampered by contamination problems. The extent of these problems is inversely related to DNA concentration and target fragment size and concern (i) sample contamination, (ii) laboratory surface contamination, (iii) carry-over contamination, and (iv) contamination of reagents.

**Methodology/Principal Findings:**

Here we performed a quantitative evaluation of current decontamination methods for these last three sources of contamination, and developed a new procedure to eliminate contaminating DNA contained in PCR reagents. We observed that most current decontamination methods are either not efficient enough to degrade short contaminating DNA molecules, rendered inefficient by the reagents themselves, or interfere with the PCR when used at doses high enough to eliminate these molecules. We also show that efficient reagent decontamination can be achieved by using a combination of treatments adapted to different reagent categories. Our procedure involves γ- and UV-irradiation and treatment with a mutant recombinant heat-labile double-strand specific DNase from the Antarctic shrimp *Pandalus borealis*. Optimal performance of these treatments is achieved in narrow experimental conditions that have been precisely analyzed and defined herein.

**Conclusions/Significance:**

There is not a single decontamination method valid for all possible contamination sources occurring in PCR reagents and in the molecular biology laboratory and most common decontamination methods are not efficient enough to decontaminate short DNA fragments of low concentration. We developed a versatile multistrategy decontamination procedure for PCR reagents. We demonstrate that this procedure allows efficient reagent decontamination while preserving the efficiency of PCR amplification of minute quantities of DNA.

## Introduction

Analysis of minute quantities of DNA via PCR is a challenge in various fields. Forensic (for reviews [1], [2]), ancient DNA (for review [3]), environmental and conservation genetic studies (for review [4]) as well as analysis of DNA in processed food [5] deal with poorly preserved biological material in which DNA is often highly degraded, thus calling for highly sensitive amplification. Moreover, pathogens in clinical specimens can be detected and identified via PCR assays, (e.g., [6]). When only few initial target molecules are amplified via highly optimized and sensitive PCR procedures, contaminating DNA becomes a major problem since even low copy contamination will be amplified leading to false-positive results. As acknowledged by Green et al. [7] and Rasmussen et al. [8], high-throughput sequencing studies of ancient genomes are not exempt from this problem, since the first experimental steps do not discriminate between endogenous and exogenous contaminating DNA molecules. When the sample nucleic acid is being investigated for medical diagnostic or forensic reasons, the impact of false-positive results can be far-reaching. The problem of contamination is often underestimated and the greatest danger might not be contamination itself but rather ignoring or neglecting it. Indeed, low copy number DNA analysis and its pitfalls are currently subject of a highly charged debate in the science and law-enforcement communities [9]. Therefore, reliable and efficient decontamination methods need to be developed for all analysis steps, from DNA extraction to PCR analysis, including PCR reagents.

Contaminating DNA can come from several different sources and is a problem when the contaminating DNA originates from the same species or genus as the sample itself. Any sample containing very little DNA is at risk of being contaminated with exogenous DNA from the same species. For instance, plant and pollen samples are at high risk of being contaminated with air-borne pollen. Forensic and clinical samples can be contaminated with the DNA of anyone who gets in direct contact with the specimen. Moreover, clinical specimens to be analyzed for microbial infections can be contaminated with hospital- and or laboratory-derived bacteria. Finally, animal samples (such as faeces, old tissue samples or archaeological/palaeontological bones) and processed food can be contaminated by food- or pet-derived DNA. Different contamination sources require different methods for elimination. (1) Contamination of specimens with exogenous DNA can occur prior to and during handling. Contamination during handling should be avoided by wearing gloves and, if necessary, whole-body protection suits. This practice is routine for sampling at crime scenes, but should also be applied to clinical, food, wildlife and archaeological specimens whenever possible [10], [11]. (2) Contamination of laboratory surfaces and devices with DNA from various sources can occur at any stage of the analysis and can be avoided by using clean rooms and decontamination methods, such as UV-irradiation and bleach treatment. (3) Amplicons are produced at very high copy number during PCR (up to 10^13^ molecules/PCR) and thus constitute a serious threat to any diagnostic, forensic or palaeogenetic analysis since they are identical to the target molecules and will be amplified with high efficiency as carry-over contamination [12]. (4) Contamination of PCR reagents and DNA extraction kits with bacterial DNA is a major problem when broad-range primers are used for the detection in clinical specimens of bacterial consensus DNA sequences, such as bacterial 16S DNA, (e.g., [13]). (5) Commercial PCR reagents [14] may be contaminated with DNA from humans and domestic animals. (6) Finally, even consumables and cotton swabs can be contaminated with human DNA [15], [16], [17].

Elimination of reagent contamination, either that introduced by the experimenter, including carry-over contamination, or arising from reagent production, presents special challenges as it requires decontamination agents that must be highly effective without affecting the efficiency and sensitivity of the PCR. Degradation by uracil-N-glycosylase of DNA synthesized in the presence of dUTP replacing dTTP is efficient for the elimination of carry-over contamination [18] but cannot be applied to native contaminating DNA. Over the years, various methods have been proposed to decontaminate reagents, such as UV- [19], [20], [21] [22] and γ-irradiation [23], isopsoralen and 8-methoxy-psoralen treatment in combination with long-wave UV light [24], [25], [26], [27], [28], hydroxylamine hydrochloride [29] or ethidium monoazide treatment [30], treatment with exonuclease III [31] or endonucleases such as DNase I [32], [33], [34], [35] or restriction enzymes [36], [37], [38], [39], [40], and finally autoclaving [15]. These methods have been tested individually and inconsistent decontamination results have been reported for all of them [19], [20], [41], [42], [43]. Furthermore, most of the decontamination procedures lead to decreased performance of the *Taq* polymerases [43]. Most importantly, these treatments were not effective for eliminating very low-molecular-mass DNA fragments (shorter than 200 bp), the main substrate of ancient and often forensic DNA analyses (e.g., [44], [45]).

Analysis of minute quantities of DNA requires the use of powerful methods. It is important that the PCR conditions are fully optimized in terms of specificity and efficiency and that nothing interferes with the detection of rare or unique molecules. Fluorescence-based quantitative real-time PCR (qPCR) highly facilitates such optimization (e.g., for a recent review see [46]). First, it allows direct measurements of the PCR efficiency. Maximal efficiency favours detection of very rare target molecules. Second, it can allow detection of parasite products generated during PCR, such as primer-dimers. This is important since formation of primer-dimers exhausts the primer pool and interferes with the amplification and detection of small numbers of initial molecules [47]. Primer-dimer detection requires the use of fluorescence dyes interacting with any double-stranded DNA, e.g., SYBR Green I® dye. In contrast, the various detection methods that use sequence-specific probes such as TaqMan and Scorpion probes and other formats [48], [49] are not as useful since they do not allow detection of these dimers that are effectively interfering with PCR whether they are detected or not. Third, forensic, archaeological, food and faeces specimens often contain polymerase inhibitors that prevent or delay the amplification reaction in a fluctuating manner thus reducing the ability to detect rare molecules. Quantitative PCR (qPCR) allows the measurement of the inhibition strength of the sample [50]. This is important since suboptimal PCR conditions can cause fluctuations in the ability to detect rare molecules. When contaminating and authentic molecules have similar low abundance, fluctuations in the detection ability can lead to confusion between them. Reliable detection of authentic but rare target molecules requires reproduction via several independent PCRs. It is important, however, to perform a sufficient number of negative controls compared to sample amplifications using the same reagent lots to ensure against low-level contaminants. The minimal number depends on the overall number of sample amplifications performed and the number of positive results obtained with these amplifications and can be estimated using statistical tools (see below).

Here, we have revisited various decontamination methods for reagents and used qPCR to quantify the efficiency of the different treatments for target DNA fragments of various sizes. We used SYBR Green I detection in combination with one of the most sensitive quantitative real-time PCR formats, the LightCycler® Instrument (Roche Applied Science, Mannheim, Germany) with a higher signal-to-noise ratio compared to other real-time PCR systems and fewer unwanted PCR products due to rapid cycling. Amplification was carried out for 60 cycles to allow for complete amplification of single molecules and the accurate detection of primer-dimers. We identified the most effective treatments for different components of the qPCR mixtures, combined them and achieved complete elimination of reagent contaminants while preserving the efficiency of the PCR. This decontamination procedure proved to be superior to other strategies.

## Results and Discussion

### Elimination of carry-over contamination

Carry-over contamination with products of previous PCR and cloning steps is one of the most serious threats for the generation of reliable results from minute quantities of DNA and also prevents the reliable evaluation of other contamination sources. The amplification and cloning of even a very small number of initial molecules produces up to 10^13^ molecules that are all identical and indistinguishable from those targeted. These contaminants may be carried over from previous amplification reactions due to aerosolization when the cap of a microtube is opened, and subsequent contamination of gloves, pipetting devices, laboratory surfaces, door knobs, handles of refrigerators and freezers, etc., in addition to reagents. This problem is exacerbated when semi-nested and nested PCR protocols are used. Carry-over contamination can be limited using dedicated devices, physical separation of the different experimental steps and stringent experimental procedures [51]. Used alone, these methods cannot guarantee complete protection [44], even when used in contained laboratories. Indeed, DNA is mostly spread by the experimenters who can be repeatedly contaminated by previous PCR and cloning products. These products can remain on many surfaces for long periods if they are not systematically identified and decontaminated after each potential contact with PCR products.

Incorporation of dUTP during PCR allows for elimination of amplicons from previous PCR and cloning steps when using uracil-N-glycosylase (UNG) [18]. We have optimized this system for quantitative real-time PCR of heavily degraded DNA (UQPCR) [12]. The efficiency of UQPCR requires a highly active UNG that is thermolabile to allow subsequent heat-inactivation of the enzyme during the PCR. We previously used *E. coli* UNG from Invitrogen (St. Louis, USA), whose thermolability was not sufficient to avoid degradation of PCR products following PCR completion when the reactions were left overnight at room temperature [12]. For higher flexibility, we sought to test the efficiency of carry-over prevention with various thermolabile UNGs from different suppliers. Using a 103-bp long PCR amplicon containing either dU or dT, we compared the activity of the widely used UNG from a marine bacterium (Roche Applied Science, Mannheim, Germany) with the UNG extracted from cod (*G. morhua*; Biotec Marine Biochemicals, Norway) at the concentration recommended by each supplier. CodUNG degraded specifically 99.96±0.04% of the dU-containing amplicons whereas the bacterial UNG degraded only 92.87±0.53%. Thus, codUNG is roughly 100 fold more efficient than the bacterial UNG and is as efficient as the previously used *E. coli* UNG [12]. Since lot-to-lot variations are possible, the efficiency of the enzyme should be regularly tested using fragments in the size and AT content range of the fragments amplified.

### Laboratory surface decontamination

In addition to carry-over contamination, environmental DNA of bacterial, human and animal origin results in widespread contamination of working surfaces, equipment and experimenters. In numerous publications and on various websites of ancient and forensic DNA facilities, the following recommendations for elimination or prevention of contaminating DNA can be found: (1) irradiation of objects with UV light; (2) wiping of objects and equipment with hypochlorite solution (bleach); (3) wiping and rinsing of objects and equipment with DNA away® (Molecular Bioproducts, San Diego); (4) the experimenter taking a shower before starting a new experiment (soap, i.e., solutions containing c. 1% of anionic and non-ionic surfactants); (5) an air shower before entering the clean room. The efficiency of these agents and procedures for the elimination of short DNA fragments smaller than 200–100 bp, however, have yet to be evaluated in a systematic way. We tested the decontamination efficiencies of those five decontamination procedures as well as that of (6) treating surfaces and equipment with copper-bis-(phenanthroline)-sulfate/H_2_0_2_ solution, “CoPA solution”, patented as carry-over prevention agent (US patent n° 5858650). We investigated the efficiency of wiping, rubbing and rinsing methods for decontamination of DNA attached to objects, using latex gloves as these are likely to be the main contamination-spreading agents.

UV-irradiation damages mainly double-stranded DNA via the formation of pyrimidine-pyrimidine photoadducts and cyclobutyl pyrimidine dimers, oxidization of bases and the introduction of single-strand breaks (SSB) and double-strand breaks (DSB) (for review [52]). The main lesions, pyrimidine-pyrimidine photoadducts prevent amplification since the *Taq* polymerase stalls at these lesions thus “neutralize” the DNA as a polymerase template. Although irradiation with UV-light of 254 nm is a simple method, it is only effective in limited conditions since the extent of DNA modification by UV light decreases with the square of the distance between the UV light source and the irradiated agent. Hence, decontamination of UV-irradiated areas is restricted to surfaces that are close to the UV bulb and is inefficient for decontamination of entire working areas, especially when UV bulbs are fixed at distance above these surfaces, such as atop containment hoods or on the ceiling. Moreover, DNA contaminants on laboratory equipment were found difficult to decontaminate with UV presumably because it is less efficient with dry DNA [15], [45].

Using qPCR we compared the efficiency of these methods on the degradation of a 107 bp long fragment in conditions that mimic low-level contamination by a PCR product. Serial dilutions of the DNA fragment solution were applied to 1 cm^2^ squares of powder free latex gloves, which were then treated by the various methods as described (see Methods S1). QPCR quantification of the recovered DNA shows that wiping, rinsing and soaking with bleach or CoPA solution are the most efficient decontamination treatments, and to a lesser degree UV treatment at high doses and short distances (i.e., at a distance of 10 cm or less for one hour) (Table 1). This is in agreement with previous results showing that exposure of DNA to sodium hypochlorite prevented PCR amplification of a 76 bp amplicon [53]. Wiping with DNA away® or with detergent removes roughly two thirds of surface-attached DNA. These treatments are thus a second choice for equipments that cannot be treated with bleach or “CoPA solution” due to their corrosive properties and should give good results in combination with short wave UV light irradiation at a short distance (at 10 cm maximum). It is also likely that it is better to substitute DNA away® by RNAse away® Molecular Bioproducts, San Diego, CA, USA) since the latter contains a higher concentration of the same effective agent according to the supplier (Molecular Bioproducts, USA; pers. comm.). Finally, air showers proved to be useless.

**Table 1:** Elimination of surface contamination.

	Air shower	Water	Detergent	DNA away®	UV	CoPA solution	Bleach
% deg	0	50,3	70,9	62,3	95,6	99,5	99,4
SD	45,5	34,5	8,9	16,9	5,6	0,5	1,2

Degradation (average percent of degradation  = % deg; SD  =  standard deviation) of the 107 bp rp49 amplicon by different agents of surface decontamination. Control experiments included an untreated PCR control and a control for recovery efficiency (water) that was subjected to the same treatment as the treated samples except that water was used as an agent. The other agents used to treat a defined quantity of DNA on the glove squares were air shower, detergent, DNA away®, 1,45 J/cm^2^ of UV light (standard UV light bulbs (254 nm) at 10 cm distance for one hour corresponding to a measured energy of 1,45 J/cm^2^), CoPA solution and bleach. DNA recovery after treatment with the various decontamination agents was measured using qPCR and was standardized by correlating it to the recovery efficiency quantified in the water control: During qPCR, the Ct (crossing point at threshold) is a linear function of the logarithm of the initial template quantity in the reaction. The extent of template degradation was therefore deduced by plotting the Cts of the treated titration series against a non-treated standard range, and quantifying the actual amplifiable template remaining in the reactions. Decrease in the quantity of the initial target molecules was attributed to both loss of DNA or degradation of DNA due to the treatment.

It should be mentioned that we wiped the glove pieces only softly with the different agents to distinguish the effects of the different treatments on the dilution of the attached DNA. Clearly, a normal decontamination procedure should be performed with more vigorous and extensive wiping and thus should be more efficient than achieved here.

In conclusion, it appears a generous treatment with bleach or CoPA solution is best to decontaminate surfaces, gloved hands and compatible equipment when performing contamination-sensitive experiments and also when in contact with PCR products that could contaminate subsequent sensitive experiments.

### Reagent contamination, low-level contamination and reliability assessment

The third major source of contaminating nucleic acids are reagents for DNA extraction and PCR. These reagents can be contaminated with DNA from various sources: human DNA, bacterial DNA and DNA from domestic animals. Plant-derived DNA could potentially be another contamination source but has not been analyzed here. Human DNA can be introduced by experimenters at any step along the production chain and may lead to erroneous results in forensic and human ancient DNA studies (e.g., [54], [55], [56]). PCR reagents, in particular the bacterially derived *Taq* DNA polymerase, can be contaminated with bacterial DNA, (e.g., [57]). Finally, we and others found that DNA from domestic animals such as cattle, pig and chicken commonly contaminates commercial PCR reagents [14]. Indeed, domestic animals are used as a ubiquitous and abundant source for the production of stabilizing agents, such as bovine serum albumin (BSA) and gelatin, as well as for the production of nucleotides [14]. These contaminants, present at very low copy number, may remain undetected unless rigorous and stringent experimental procedures are used. These include, as discussed before, the use of highly efficient optimized PCR conditions and the performance of a high number of non-template control (NTC) reactions, i.e., negative controls without sample. Low-level contamination can easily be overlooked when low numbers of NTCs are performed. A statistical approach relying on the binomial distribution allows to determine the lowest contamination level that can be ruled out with 95% confidence considering the proportion of contaminated NTCs in an experiment, and the number of all-negative controls that are necessary to exclude a certain theoretical contamination level (Figure S1) [58]. For example, if 100 NTCs have been performed and one yielded a PCR product, then one can assume with a 95% confidence level that the total contamination level of the PCRs is lower than 4.7%. In contrast, if only 30 NTCs are performed, even without yielding a single positive PCR, then one can only assume that the total contamination level is lower than 9.5% of the PCRs (Figure S1).

We performed an extensive survey of the occurrence of reagent contamination using conditions tailored to prevent other sources of contamination: a high containment facility, physical separation between pre- and post-PCR working areas and enzymatic carry-over prevention with UQPCR [12]. Stringent experimental procedures are also required to minimize the dispersal of aerosol-borne DNA or DNA that is attached to gloves. We monitored contamination in approximately 1,500 blank controls using equine, reindeer, cheetah or mammoth primers for mitochondrial sequence during our analyses of ancient *Equus*, *Rangifer, Acynonix* and *Mammuthus* bone remains of various origins and ages (between 1,000 and 100,000 years). We successfully amplified and sequenced numerous samples of all of these species, whereas not a single contaminated NTC was ever obtained in parallel. This shows that our procedures prevent the occurrence of carry-over contamination to the highest extent possible. It also shows that PCR reagents are not contaminated with DNA from these species. When using mitochondrial DNA primers and various *Taq* polymerases, however, we noticed contamination of blank controls with bovine and porcine sequences. In particular, using the FastStart DNA Master^PLUS^ SYBR Green mix (Roche Applied Science, Mannheim, Germany), 41 out of 62 NTCs yielded bovine amplicons of 153 bp that can be attributed to contamination of BSA contained in the kit with trace quantities of bovine DNA, most likely around one molecule per 10 µl of reaction volume. When using other *Taq* polymerases in combination with a home-made PCR mix (see Material and Methods), the contamination rate was generally lower than one molecule per reaction, but it was high enough to yield 151 positives out of 1,170 NTCs in experiments performed over several years. Moreover, when using AmpliTaq Gold (Applied Biosystems, CA, USA), we increased the number of amplification products in the negative controls when increasing the quantity of AmpliTaq Gold in the PCR while keeping the amplification efficiency at roughly the same level. This shows that not only dNTPs may be a source of reagent contamination as proposed [14], but that enzymes can also be contaminated with DNA from domestic animals. The detected contamination often occurred in a lot-dependent fashion and lead to amplification products in about 13% of our blank controls (between 9 and 66% depending on the reagents used). This can equal the average success rate of delicate studies, such as ancient DNA analyses from poorly preserved bone material. Therefore, there is a high risk that the sequences obtained from this type of contamination can be mistaken as authentic positive results.

We sequenced a number of PCR products from the NTCs and found the bovine sequences to correspond to European cattle. The display of the sequences (Figure S2A) using median joining networks [59] showed a sequence distribution close to the one of present day European cattle [60] and found in some studies of Neolithic and Bronze Age cattle (e.g., [61]) and Palaeo- and Mesolithic aurochsen (e.g., [62]). Similarly, for pig mitochondrial DNA sequences, contamination revealed a diversity comparable to the extant Eurasian diversity [63] (Figure S2B). Even if data are reproduced within and between laboratories, the obtained sequences can be contamination-derived since the reagents can carry the same sequences depending on the batch of the reagent and even across batches and suppliers, as seen for bovine mitochondrial DNA. Therefore it is crucial in the case of samples that yield the same sequences as the reagent contamination to have an amplification success that is much higher than the contamination “noise” and a sufficient number of negative controls performed (Figure S1).

The reliability of PCR amplifications may be assessed by using a statistical test to compare the proportion of successful amplifications in sample and control PCR, in order to determine whether the sample PCR success rate is significantly different from amplification rate due to contaminants. As the typical number of successful attempts in conventional genetic studies with degraded samples is often low, Fisher's exact test is generally the most appropriate for this comparison. Table S2 displays the minimum numbers of independent sample PCRs and minimum success rates necessary to validate a result according to Fisher's exact test, depending on a range of false positive rates observed in NTCs. For example, if only 5 NTCs are performed and none gives rise to a product, a sample analyzed simultaneously would have to yield a PCR product in two out of two attempts, or, if one PCR attempt fails, three out of four attempts must be positive in order to be considered statistically reliable. This stresses the importance of performing a large number of NTCs as well as replicates to reach a 95% confidence level in PCR amplification results from difficult samples, when sample sequence cannot be distinguished from known contaminants. Of note, as the point of the test is to compare the proportion of successful amplifications of NTCs and samples, samples that do not yield a product cannot be considered as negative NTCs, and data from series with positive NTCs must not be discarded, except in situations where clear high-level contamination rate is observed, which would be indicative of contamination from another source, i.e., carry-over contamination.

When using commercial reagents without special decontamination procedures, 151 positive NTCs were obtained out of 1170. Such a level of contamination can easily remain undetected when a small number of NTCs are considered. As it is not practical to perform a large number of NTCs with every sample, one option is to analyze several samples simultaneously or, at a broader scale, to pool all NTC data obtained over many experiments with a given reagent batch. Many sample PCRs would then be compared to the same NTCs: these multiple comparisons increase the risk of obtaining statistically significant differences by chance and corrections are necessary to ensure a 95% confidence level. The Bonferroni correction is classically used and aims at excluding such false positives. Table S2 displays the results of this correction applied to example situations where either 10 or 50 samples are compared to the same NTCs. For instance, if only 5 NTCs are performed and 10 samples are analyzed in parallel, data from a sample can be trusted with 95% confidence only if the corresponding PCRs are replicated 6 times out of 6 attempts. When 50 samples are compared to our entire pool of 1170 NTCs (see above), a sample can be trusted only when replicated 4 times in 4 PCR attempts. As the number of samples increases, a significant difference between the success rate in samples and NTCs becomes virtually impossible to reach even when the number of NTCs is huge. The Bonferroni correction indeed has the disadvantage of being conservative, and excludes false positives at the expense of a certain number of false negatives. A trade-off between false positives and false negatives can be obtained using the Benjamini Hochberg false discovery rate (FDR) approach [64]. P-values must be calculated for all samples analyzed and ranked to determine the samples to be considered while accepting a certain FDR. Additional replications on this smaller sample set could then be performed to confidently exclude the false positive results.

When difficult and precious samples are analyzed using species for which contamination is ubiquitous, we would recommend systematic recording of all experiments performed with a given reagent batch and statistical estimation of samples reliability. To ensure authenticity of the data with a 95% confidence level for species that potentially contaminate PCR reagents, the DNA in the samples analyzed must be well-preserved, or, if DNA preservation is poor, a large number of replications and controls may be necessary. Thus, for most palaeogenetic analyses of ancient bones, and in particular those of humans and of animals used for reagent preparations (e.g., cattle and pigs, maybe chicken [14]) that reveal a population distribution similar to that of modern populations, it is clear that much stricter criteria than those currently used in the field are required to claim with confidence the authenticity of a sequence that is identical to that commonly found in reagents. In particular, it appears at least as important to report the numbers of PCR attempts with and without samples performed as to replicate a subset of the analysis in another laboratory.

We analyzed another potential property used to discriminate between modern contaminating and authentic ancient DNA sequences, the size of the amplifiable fragments. Indeed, long amplicons have been usually considered as an indication of modern DNA contamination, while samples yielding only short amplicons are often thought to correspond to authentic ancient DNA (e.g., [3], [65], [66]). We therefore investigated the fragment length of the contaminating DNA molecules. When using primer pairs amplifying fragments of either 555 bp, 702 bp, or 1107 bp, no amplification product was obtained in negative controls with the FastStart DNA Master^PLUS^ SYBR Green mix that yielded a contamination rate of 66% when amplifying a 153 bp fragment (see above). Thus, DNA contaminating reagents appear to be also of relatively low molecular weight, and small fragment size cannot be used as a reliable authentication criterion in ancient DNA studies. This is in agreement with results from the analysis of whole genome sequencing experiments [67] and is particularly true when the PCR system is less reliable and more mutagenic than the UQPCR procedure [68]. It is reasonable to assume that DNA traces in serum albumin and gelatin are degraded since the DNA in these reagents should originate from lysed blood cells in the serum or hydrolyzed animal tissues. Human contaminating DNA is likely to stem from dead and often dried cells in which DNA is degraded into short fragments [15], [69].

Another criterion to discriminate endogenous, ancient DNA fragments and exogenous, contaminating modern DNA fragments is based on the specific distribution of deaminated cytosines in ancient DNA fragments. This criterion has been recently used to assess the authenticity of next generation sequencing data [67], [70]. Deamination was shown to occur preferentially near the end of the DNA fragments [67], [70]. One could argue, however, that depending on their origin and their age, some contaminants could have been deaminated as well and it will be necessary to perform more studies to assess the reliability of this criterion. Furthermore, deamination of the ends of the molecule cannot be assessed using standard PCR approaches because PCR primers hybridize at positions nested in-between the ends of the fragments analyzed.

In light of these results and analyses, it was important to develop a system of physico-chemical destruction of the contaminating DNA in PCR reagents. Since we found commercial amplification kits to be heavily contaminated with exogenous DNA, and a home-made PCR mixture with chemically synthesized dNTPs to be moderately contaminated, we explored other approaches to further reduce contamination levels.

### Degradation of double-stranded DNA by γ-irradiation

γ-irradiation is commonly used to inactivate pathogens such as bacteria and viruses. Inactivation of viruses requires only a few double-strand breaks in genomes that are several kilobases long. Since in the ancient DNA field or in forensic analyses, small DNA fragments are targeted for PCR amplification, due to the degradation of the target DNA, the contaminating DNA must be broken into much smaller fragments for the decontamination to be efficient. γ-irradiation of DNA in a dry state produces mainly double-strand breaks (DSBs) in contrast to γ-irradiation of aqueous solutions that produces in addition high amounts of OH radicals, which induce single-strand breaks (SSBs) in DNA, (e.g., [71]). The irradiation of DNA in solution induces several orders of magnitude more breaks than of DNA in a dry state. For dry DNA at 25°C, 5.7×10^−11^ SSBs and 3.2×10^−12^ DSBs are induced per Gy and Dalton of DNA whereas for DNA in aqueous solution at 25°C, 1.1×10^−7^ SSBs and 5.4×10^−9^ DSBs are induced [72]. This indicates that hydroxyl radical production is the key factor in the decontamination of DNA in solution. Based on these values, the irradiation with 1 kGy should induce about 1–2 SSBs in a 50 nucleotide-long DNA fragment in aqueous solution. γ-irradiation at a dose of 1.5 kGy was proposed to decontaminate carry-over PCR products as small as 280 bp [23]. Since γ-irradiation is a clean, easy to handle physical agent that is available in most hospitals to ensure sterilization and pathogen inactivation, we measured its ability to decontaminate water and PCR reagents.

**Figure 1:**
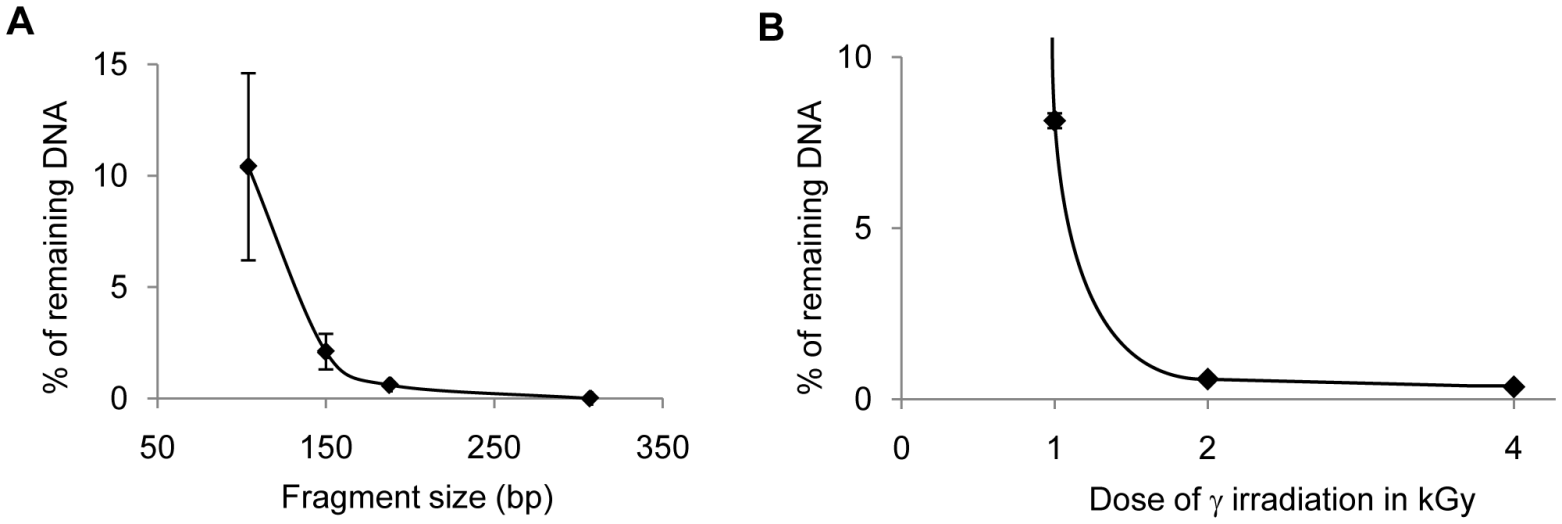
Degradation of DNA by γ-irradiation. **A. Size-dependence of degradation by γ-irradiation of phage λ DNA.** Phage λ DNA in water was γ-irradiated with 1 kGy and qPCR quantification of fragments of various size (104 to 307 bp) was performed. The average of the percentage of remaining DNA is plotted as a function of amplicon length. **B. Effects of the dose of γ-irradiation on the degradation of a 73 bp DNA fragment.** Phage λ DNA in water was γ-irradiated with 1, 2 or 4 kGy and qPCR quantification of a 73 bp fragment was performed. The average of the percentage of remaining DNA is plotted as a function of the irradiation dose.

We performed γ-irradiation tests to evaluate the minimal irradiation dose necessary to degrade the smallest contaminating fragments, typically target DNA fragments of 150 bp or less. First, we established the size-dependence of the degradation efficiency of DNA fragments via irradiation using qPCR on a DNA concentration range. γ-irradiation with 1 kGy of λ-DNA in water and subsequent amplification with primer pairs amplifying a 307 bp, a 188 bp, a 150 bp, and a 104 bp DNA fragment, showed that while the 307 bp and the 188 bp targets were totally degraded, only 97.94% of the 150 bp target and 90% of the 104 bp target were degraded (Figure 1 A). We then focused on identifying conditions suitable to degrade a small 73 bp target because amplification of such small fragments is often necessary when analyzing highly degraded samples. After irradiation with 2 kGy or more, the concentration of 73 bp and larger DNA target molecules was reduced by over 99% (Figure 1 B). Thus, our results show that γ-irradiation with 2 kGy or more eliminates most DNA molecules, even very small fragments that might contaminate the water used in DNA extraction, purification or amplification steps.

However, when a similar experiment was carried out on DNA diluted with the reagents used in the preparation of the qPCR mix, we found the decontamination efficiency of γ-irradiation by 4 kGy to be severely decreased: the quantity of templates amplifiable with the 73 bp primer pair was reduced 10-fold at most when DNA was diluted with most of the reagents (glycerol, serum albumin, detergents such as Lubrol; Table 2). This short DNA fragment was even not significantly degraded at all when irradiated in a dNTP- or 2-aminopropanediol-containing solution. This can be attributed to the scavenger effect: most organic molecules act as scavengers against the free radicals produced by γ-irradiation in aqueous solutions by absorbing the oxidative effects of the radicals and shielding DNA molecules [73]. We conclude that γ-irradiation is not a reliable means to decontaminate qPCR reagents other than water. We therefore limit the use of a 5 kGy γ-irradiation to the decontamination of water used at all experimental steps.

**Table 2:** γ ray-induced Degradation of DNA in various buffers.

	Water		2-amino-propanediol		Glycerol		HSA		Lubrol		dNTPs	
	% deg	SD	% deg	SD	% deg	SD	% deg	SD	% deg	SD	% deg	SD
UV	99,73	0,35	99,40	1,05	99,68	0,59	98,78	0,78	99,80	0,14	19,51	6,26
γ	99,97	0,05	0		66,93	5,64	94,63	1,05	33,56	7,08	23,27	11,07

Average percentage of degradation (% deg) of 25 ng/µl of λ DNA by γ-irradiation with 4 kGy and UV irradiation for 10 minutes in a UV crosslinker (see Material & Methods) in stock solutions of various reagents. Water was complemented with 2.5 M 2-aminopropanediol, 50% glycerol, 10 mg/ml horse serum albumin (HSA) in water, 10% Lubrol in water, 20 mM dNTPs (dATP, dCTP, dGTP), 40 mM dUTP, followed by amplification of a 73 bp DNA fragment. (SD = standard deviation).

Finally, we also found that the *Taq* DNA polymerase does not tolerate irradiation with more than 1 kGy without significant loss of efficiency. Since *Taq* DNA polymerase, which is extracted from bacteria and stabilized with proteins extracted from animal tissue such as BSA and gelatin, is a major contamination source for bacterial, animal and human DNA among PCR reagents, it constitutes a critical reagent to be decontaminated.

### UV-irradiation

UV irradiation is not only often used in surface decontamination, it has also been proposed as a mean to decontaminate PCR mixtures [19], [20], [41], [74]. These studies showed that UV irradiation decreases by 1,000 to 100,000-fold the carry-over contamination present in a single PCR tube since DNA is damaged, i.e. “neutralized” for PCR. Inhibition of PCR amplification of DNA molecules by UV light was shown, however, to depend on the distance from the UV source [75] and on the molecular weight of the DNA molecules, small DNA fragments (151 bp) being poorly “neutralized” [45]. Plastic reaction tubes were found to inhibit PCR after UV irradiation [76]. Finally, dNTPs, which strongly absorb UV (254 nm), were found to protect DNA against UV damage, thus requiring a much longer UV-irradiation time to “neutralized” DNA molecules [41], [75]. Therefore, several authors found UV irradiation to be inefficient in damaging contaminating DNA to prevent its PCR amplification [24], [26], [43], [77], [78]. Moreover, primers and *Taq* DNA polymerase were found to be UV-sensitive, reducing the sensitivity of the PCR amplification up to 10-fold [19], [42]. Thus, irradiation with UV-light cannot be applied as a general prePCR method since it will damage the PCR reaction mixture if all components including *Taq* DNA polymerase and primers are initially added [41], [75]. To explore the usefulness of UV-irradiation at 254 nm, we performed a systematic test of its efficiency with the various components of PCR mixtures, in particular those that are refractory to decontamination with γ-irradiation.

#### Inactivation of double-stranded DNA with UV-irradiation

First, we tested the efficiency of 254 nm-UV irradiation to “neutralized” dsDNA in a similar fashion as described for γ-irradiation, by quantifying the remaining templates in a UV-irradiated bacteriophage λ DNA aqueous solution using qPCR with the aforementioned three different primer pairs. We used a Stratalinker® UV Crosslinker 2400 device (Stratagene, Cedar Creek, USA) and later a Spectrolinker XL 1500 UV crosslinker (Spectronics Corp. Westbury, NY, USA), which both deliver an overall energy whose measurement by the internal measuring cell depends on the coating of the walls (see Methods S1). The efficiency of amplification prevention of the target DNA molecules was crucially influenced by the plastic material of the irradiated tubes and the distance of the irradiated tube from the UV light source.

We observed that the plastic recipient in which UV-irradiation is performed strongly influences the decontamination efficiency, as many plastics used for molecular biology containers absorb UV rays. DNA inactivation was not very efficient when using standard 1.5-ml Eppendorf tubes: between 90 and 97% of the 73 bp fragment was “neutralized” when between 50 µl and 1 ml of 250 pg/µl solutions were irradiated in the Stratalinker for 10 minutes at 1 cm from the UV bulbs. The “neutralization” was more efficient and reproducible when performed in thin-wall clear polypropylene tubes, such as 0.2 ml PCR tubes from Abgene Limited (Epsom, UK) or Qubit 0.6 ml tubes from Invitrogen. In the latter ones, between 99 and 99.2% of the 73 bp fragment was “neutralized” when between 10 and 500 µl of 250 pg/µl solutions were irradiated in the Stratalinker for 10 minutes at 1 cm from the UV bulbs. In 0.2 ml PCR tubes the efficiency of preventing amplification was even higher since between 99 and 100% of the 73 bp fragment were “neutralized” when 10, 50 or 100 µL of the DNA solution were irradiated. This was also true when DNA was irradiated in qPCR buffer.

Attempts to irradiate larger volumes, up to 4 ml in 15 ml Falcon tubes, were unsuccessful leading to only 47% of “inactivation” of the 73 bp target (irradiation for 10 minutes at 1 cm from the UV bulbs). This low efficiency was presumably due to a combination of certain properties of the plastic ware and the shape of the tubes since better efficiencies could be achieved using a smaller volume in the same tubes. 96% of the 73 bp target were “neutralized” when only 1 ml was irradiated, and 99% when 10 µl were irradiated for 10 minutes at 1 cm from the UV bulbs. These results clearly show that UV irradiation is efficient only under narrow experimental conditions using small volumes in specific plasticware and do not support the efficacy of decontamination procedures using UV irradiation of large volumes of liquid.

**Figure 2:**
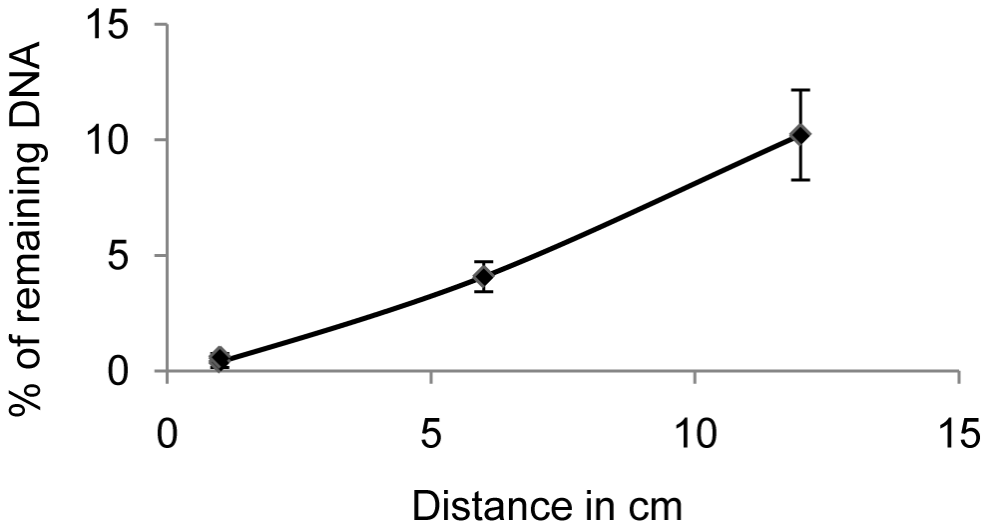
Efficiency of decontamination as a function of the distance from the UV light source. Phage λ DNA in water was UV-irradiated for 10 minutes in the UV crosslinker at 1 cm, 6 cm and 12 cm from the light bulbs and qPCR quantification of a 73 bp fragment was performed. The average of the percentage of remaining DNA is plotted as a function of the distance from the bulbs.

DNA degradation could be shown to be a function of the distance from the UV light source. When we quantified by qPCR amplification of a 73 bp fragment the DNA that had been irradiated for 10 minutes in the UV crosslinker at 1 cm, 6 cm and 12 cm from the light bulbs, the percentage of amplification prevention achieved was 99.7±0.3%, 96±0.6%, and 89±1.9%, respectively (Figure 2). Thus, the efficiency of preventing the amplification of small DNA fragments decreases rapidly with increasing distance from the UV light source, and only solutions irradiated directly under the UV light bulbs can be efficiently decontaminated. These results question decontamination procedures using UV bulbs on the ceiling or at the top of containment hoods. Using the optimal distance, the time of UV irradiation was calibrated as a function of the DNA fragment size by qPCR amplification of fragments of different length. The larger DNA targets (188 and 307 bp) were “neutralized” to 99% after 2 minutes and completely after 4 minutes. In contrast, only 97.5% of the smallest 73 bp fragments were “neutralized” after 2 minutes of irradiation, 98% after 4 minutes, 99% after 8 minutes and 99.9%, after 10 minutes.

Irradiation of DNA in dNTP-containing buffer at a final concentration corresponding to the 10 x qPCR buffer allowed efficient amplification prevention only of large DNA fragments (about 99% for a 244 bp fragment and about 96% for a 150 bp fragment) but was not efficient enough for small fragments (73 bp), with only about 90% of amplification prevention, confirming previous observations by other authors [41], [45], [75]. When irradiation was performed on DNA buffered with the 10 x qPCR buffer without dNTPs (and also without *Taq* DNA polymerase and SYBR Green I) in 100 µl aliquots, the 73 bp target DNA was “neutralized” by 99.2±0.2% indicating that it is feasible to irradiate a subgroup of the reagents of the PCR mix in a single tube. Alternatively, the same reagents can be irradiated independently as stock solutions and can be mixed afterwards with the reagents that do not resist UV irradiation (see below).

#### Effects of UV-irradiation on the PCR performance

To assess the feasibility of the UV irradiation treatment for decontamination, we irradiated the qPCR buffer and compared the PCR kinetics with non-irradiated controls. In particular, we determined the efficiency of each qPCR (see Methods S1 and [79]). After UV irradiation, the reaction kinetics of the PCR was unaltered if the irradiation was performed in the absence of dNTPs, *Taq* DNA polymerase and SYBR Green I (data not shown). In contrast, *Taq* DNA polymerase and SYBR Green I dye were found to be completely “inactivated” by UV irradiation that efficiently decontaminates small DNA fragments. SYBR Green I is a chemically synthesized compound that is used at a very low final concentration. We evaluated that the risk of contamination of SYBR Green I was low when the solution is prepared with decontaminated reagents (e.g., UV irradiated Tris buffer), when extreme care is taken to prepare it in a confined and decontaminated area, and when aliquots are used. Thus, we have not further attempted to decontaminate it by other means and only add it to the irradiated PCR buffer. We also deemed UV irradiation of the primers to be unreliable and have refrained from testing it, because both PCR performance and adverse effects of the UV treatment can vary depending on the primer sequence (e.g., number of successive thymines in the sequence). Therefore, while most qPCR reagents can be decontaminated efficiently and easily by short UV irradiation treatment, to ensure complete decontamination *Taq* DNA polymerase, primers and dNTPs have to be subjected to a different treatment when such reagents are likely to be contaminated. Indeed, these reagents are an important source of contamination because fifteen 153 bp-long bovine amplificons out of 372 NTCs were still obtained using a UV irradiated PCR mix without treating *Taq* DNA polymerase and dNTPs.

### Endonuclease treatment of double-stranded DNA

To decontaminate reagents that cannot be decontaminated by irradiation, we considered various nuclease treatments. Restriction endonucleases, although proposed by several authors [36], [37], [38], [39], [40], are not a reliable and versatile solution, since different enzymes must be used according to the sequence of the contamination product, different enzymes will show different suitability, inactivation conditions and efficiency of decontamination and some contaminant may not contain any restriction endonuclease sites. Thus, it is impossible to test rigorously their suitability. We therefore tested nucleases that display no sequence-specificity of cleavage.

#### DNase I

DNase I has been proposed as an agent to degrade contaminating DNA molecules in solutions containing irradiation-sensitive reagents, such as enzymes and primers [78] as well as to eliminate carry-over contamination prior to PCR [32], [33], [34], [35], [36], [42]. For certain applications such as amplification of trace quantities of human DNA of forensic or ancient samples, it is advisable to also decontaminate primers which might be contaminated by human DNA during production. DNase I is a DNA endonuclease that hydrolyzes preferentially double-stranded DNA and cleaves without sequence specificity but with some DNA conformation preference [80]. We observed, however, that DNase I can also degrade some primers to various extents (Figure S3). We attribute this effect to the sequence dependent stem-loop formation in certain primers that are a target for DNase I.

Another critical issue is heat-inactivation of the DNase I necessary to guarantee efficient inactivation of the DNase I prior to PCR [42], [43], [81]. This heat inactivation step is of concern since high temperature is likely to activate prematurely the hot-start *Taq* polymerases, thus increasing primer-dimer formation. Although in our hands a 10 minute-incubation of the PCR mix with DNase I at room temperature was sufficient to completely degrade any DNA present in the reaction mixture, the Hot-Start *Taq* polymerase was prematurely activated during the 10 minute 95°C DNase I deactivation step, leading to a significantly decreased PCR sensitivity (see Methods S1).

To conclude, DNase I is not a satisfactory decontamination reagent due to the degradation of certain primers and the need of extensive heat inactivation that activates prematurely the hot start DNA polymerases.

#### Heat-labile dsDNase

To avoid the negative effects of a heat inactivation step at 95°C and to ensure double-strand specificity we compared two other endonucleases: (i) the recombinant double-strand specific shrimp endonuclease® (dsDNase) isolated from *Pandalus borealis* (Biotec Marine Biochemicals, Tromsø, Norway), and (ii) a heat-labile mutant version of it (hl-dsDNase® Biotec Marine Biochemicals, Tromsø, Norway). These enzymes were reported to be 20,000-fold more active on dsDNA than on ssDNA and to have a five-fold lower activity on denatured DNA than DNase I [82]. They remove 2×10^9^ molecules of 507 bp within 2.5 minutes at room temperature in a 1 x PCR buffer of *Taq* DNA polymerase (Patent No. US 6,541,204 B2 and [82]). The dsDNase can be irreversibly inactivated at 65°C, and the new hl-dsDNase at 55°C in the presence of DTT [82]. This latter property makes these nucleases potentially superior to DNAse I because at these temperatures, it is likely that Hot-Start *Taq* polymerases are not prematurely activated and other proteins such as BSA are not denatured. dsDNase has been used to minimize contamination of buffer, dNTPs and primers prior to PCR, but not of the *Taq* DNA polymerase [83].

**Figure 3:**
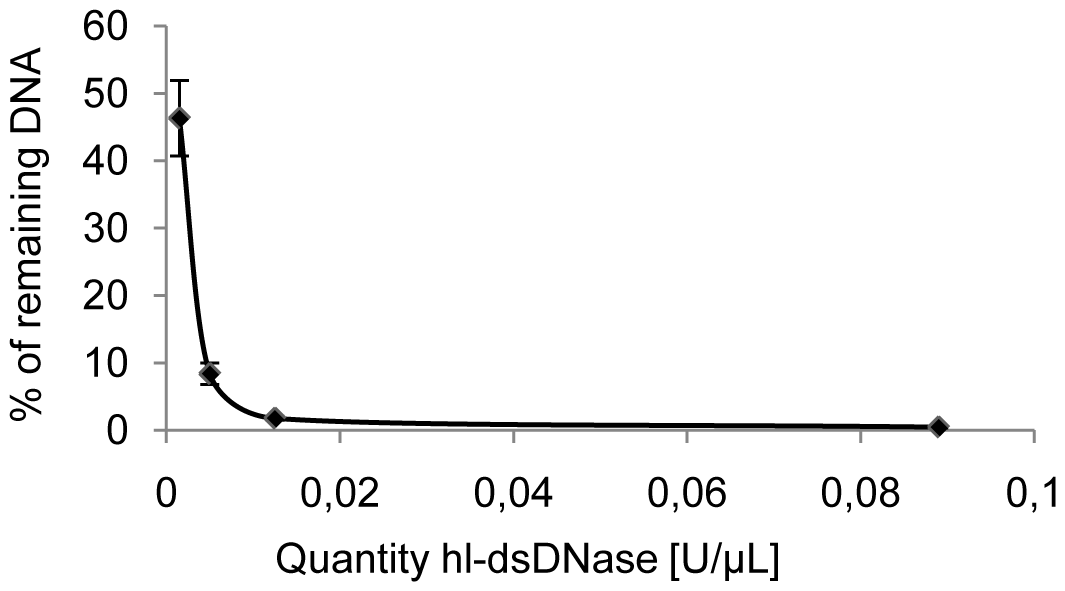
Efficiency of hl-dsDNase treatment. 1.3 pg of λ DNA was incubated with various quantities of hl-dsDNase in 90% *Taq* DNA polymerase storage buffer for 30 minutes at 25°C and qPCR quantification of a 73 bp fragment was performed. The average of the percentage of remaining DNA is plotted as a function of the quantities of hl-dsDNase.

To test the efficiency of the dsDNases as a decontaminating agent of *Taq* polymerases, primers and dNTPs and to find the minimal enzyme concentration ensuring most efficient decontamination, we evaluated the degradation of λ DNA using dilutions of dsDNAses in different buffer conditions. To decontaminate *Taq* DNA polymerase, we chose to use conditions that minimize changes in the *Taq* storage buffer composition in order to enable long term preservation of the enzyme at −20°C after decontamination in batch. The tests were carried out in the storage buffer of the FastStart® Taq DNA polymerase (Roche Applied Science, Mannheim, Germany), supplemented with 10 mM MgCl_2_, 1 mM CaCl_2_ and 1 mM DTT that are required by the dsDNases to be active or to be efficiently inactivated. The supplemented buffer corresponded to 90% *Taq* storage buffer and we also tested a further dilution to 50% storage buffer. When we compared the activity of the two dsDNAses, we found both enzymes to be 80 times less active in 50% *Taq* storage buffer and 300–400 times less active in the 90% supplemented storage buffer than in the recommended conditions of usage (see Methods S1). Therefore, we measured the degradation of a 73 bp target in 90% *Taq* storage buffer supplemented with 10 mM MgCl_2_, 1 mM CaCl_2_ and 1 mM DTT using varying quantities of hl-dsDNase for 30 minutes at 25°C (Figure 3). Most (99.5±0.14%) of the DNA molecules longer than 73 bp were degraded with at least 0.02 U/µl of hl-dsDNase and 0.1 U/µl was chosen as a standard dose to ensure reliable DNA polymerase decontamination.

We also tested the efficiency of the treatment with hl-dsDNase of dNTPs, which are not always synthesized chemically but also obtained by hydrolysis of animal tissue and therefore could be contaminated with bovine or porcine DNA (see above). Moreover, contamination with human DNA can never be excluded. Using the conditions described in Methods S1, in buffer containing 2 mM of each dATP, dCTP, dGTP and 4 mM dUTP it was possible to degrade 98% of the DNA. In contrast, hl-dsDNase was totally inhibited when the dNTP concentration was 30 mM final (6 mM each dC,G,ATP, 12 mM dUTP). Thus, dNTPs can be decontaminated at a concentration of up to 2 mM each.

In contrast to DNase I, which cannot be used to decontaminate primers, we found that hl-dsDNase does not degrade most primers, including those shown to be sensitive to DNase I treatment (BR2). Indeed, the efficiency and sensitivity of the PCR was not altered compared to the untreated control (Figure S4). None of the 30 primers we tested was degraded by treatment with hl-dsDNase. Since some primers might generate stem-loop secondary structures that are double-stranded and thus sensitive to dsDNase digestion, we nevertheless recommend to verify that their performance has not been affected by treatment with hl-dsDNase. In studies where contamination of primers by the DNA to be amplified is unlikely, decontamination of primers might not be necessary.

In conclusion, the hl-dsDNase treatment is an efficient way to decontaminate PCR reagents that cannot be decontaminated by irradiation, in particular *Taq* DNA polymerase and dNTPs. Decontamination of all reagents, including primers, is particularly important for forensic analyses and studies of ancient human remains where any reagent can be contaminated with human DNA.

#### Inactivation of the heat-labile dsDNase

We optimized the inactivation conditions of the hl-dsDNase to minimize heat damage of components of the PCR mix. To reveal potential residual activity of the enzyme, we tested the inactivation times and buffer compositions that were used to decontaminate dNTPs, primers or *Taq* polymerase comparing side by side dsDNase and its thermolabile mutant hl-dsDNase. Following inactivation by various treatments, the residual DNase activity was measured by incubation of λ DNA in optimal conditions and the degradation of 73 bp and 153 bp λ DNA target molecules was quantified by qPCR (Table 3). In *Taq* storage buffer containing 1 mM DTT, a 15 minute-incubation at 50°C was necessary to inactivate most of the hl-dsDNase whereas higher temperatures were necessary to inactivate the wild-type dsDNase. The inactivation treatment of hl-dsDNase could induce a loss of sensitivity of the PCR since it could activate some of the Hot Start *Taq* DNA polymerase prior to the beginning of the PCR. As a consequence primer-dimers could appear earlier. To prevent undesired preactivation of the hot start *Taq* DNA polymerase during the dsDNase inactivation step, we chose to use the mildest heat treatment ensuring complete inactivation (20 minutes at 50°C). Then we compared PCRs with and without hl-dsDNase treatment and inactivation. We found that it had little effect on the PCR kinetics and on primer-dimer formation. In a few instances, delay in PCR amplification was observed that was not exceeding one cycle with the various primer sets tested (ΔCt ranging from 0.09±0.1 to 0.94±0.3). For the inactivation of the DNase used to decontaminate dNTPs and primers, it is better to use harsher inactivation conditions (30 minutes at 55°C in the presence of DTT) since in the buffer used the enzymes are more thermoresistant (Table 3). This is not a problem since the corresponding PCR reagents are not particularly heat-sensitive.

**Table 3:** Inactivation of wild-type and mutant hl-dsDNase using different buffer conditions, incubation times and temperatures.

	Tris buffer						*Taq* buffer				
	55°C				60°C		50°C			55°C	
	no DTT		1 mM DTT		no DTT						
	10 min	30 min	10 min	30 min	10 min	30 min	10 min	15 min	30 min	10 min	30 min
dsDNase	98,8	99,7	99,5	100	n/d	n/d	n/d	n/d	n/d	99,9	100
	98,5	99,7	99,5	99,7	99,6	100	<80	n/d	98,5	98,5	100
hl-dsDnase	97,5	99,2	99,8	99,4	n/d	n/d	n/d	100	n/d	100	100
	98,5	99,4	100	99,8	99	100	99,2	99,6	100	100	100

The remaining activity of the endonucleases after inactivation was quantified through the degradation of λ DNA and subsequent PCR using primer pairs L9/5 (73 bp fragment; upper box) and L9/10 (153 bp fragment; lower box). The longer fragment was used as a more sensitive measurement of residual DNase activity. The table indicates the percentage of inactivated enzyme. *Taq* buffer contains 1 mM DTT. n/d, not done.

Finally, we tested whether primers and *Taq* DNA polymerase that had been treated with hl-dsDNase could be kept at −20°C after treatment by testing their performance during PCR over a period of one month. This long-term storage proved to allow optimal PCR over time, showing that this aspect of the decontamination procedure can be implemented easily.

#### Performance of the heat-labile dsDNase treatment

Since PCR reagents are commonly contaminated with bovine DNA, the efficiency of the treatment to eliminate endogenous contamination of PCR reagents was tested with a series of NTCs using a primer pair (BB3/4) targeting a 153 bp fragment of the bovine D-loop. We used PCR reagents that we found to be particularly contaminated by bovine DNA. Following decontamination of FastStart Taq DNA Master^PLUS^ SYBR Green mix (Roche Applied Science, Mannheim, Germany) and hl-dsDNase inactivation as described in the Methods S1, we verified that the efficiency of the PCR was unaffected by the treatment and we quantified the efficiency of the removal of the contaminant by performing a high number of NTCs. For the hl-dsDNase-treated reaction mix, one bovine amplification product was obtained out of 63 NTCs none of which yielded any primer dimers. In contrast, the untreated reaction mix yielded a bovine amplification product with 41 out of 62 NTCs.

Thus, the hl-dsDNase treatment of a commercially available PCR mixture that we found to be contaminated with bovine DNA in 66% of the performed blank controls decreased the contamination to 1.5% and therefore proved to be relatively efficient, but not sufficient to completely decontaminate.

### Performance of the UVD-decontamination procedure

**Figure 4:**
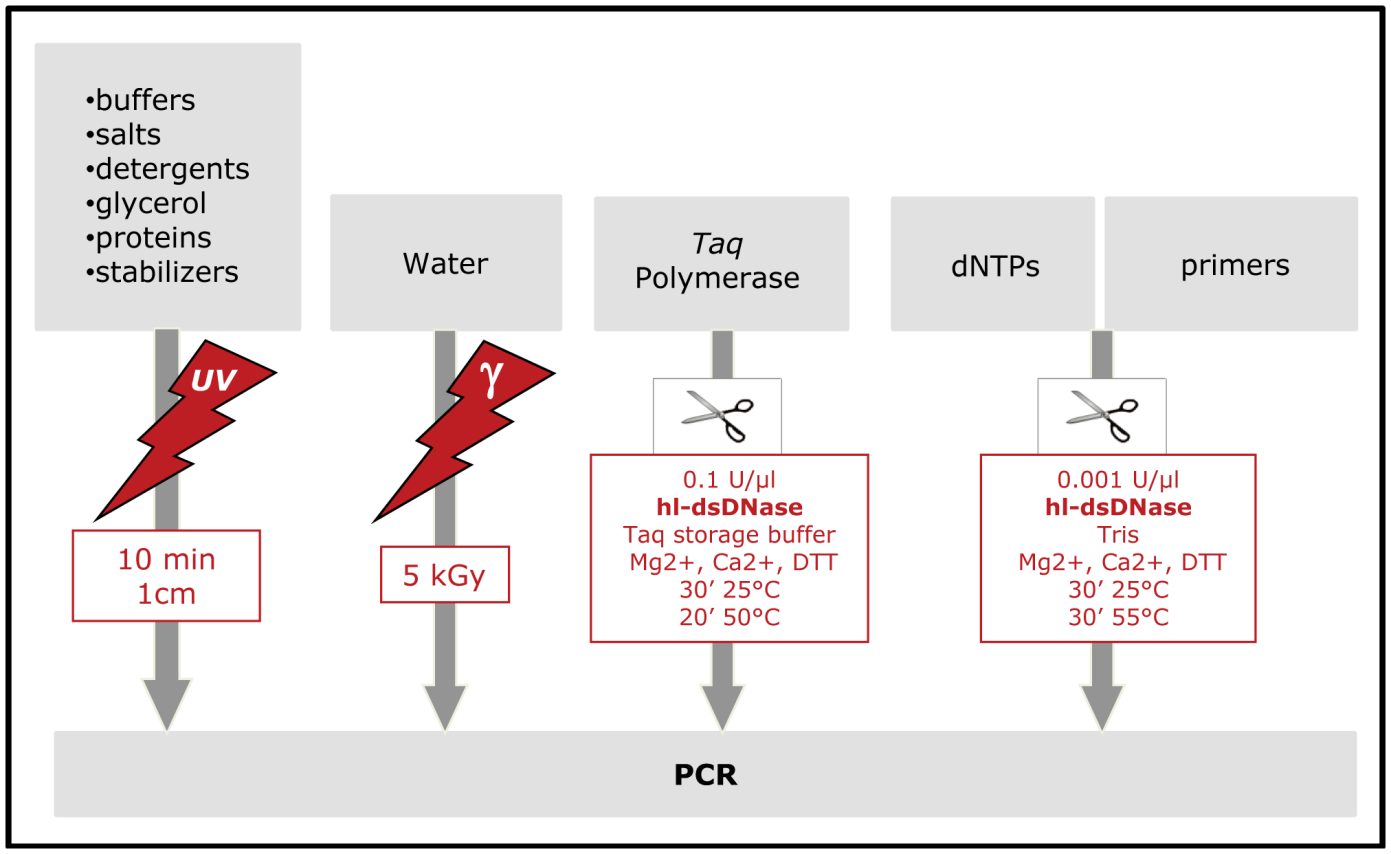
Flow chart of the UVD decontamination procedure.

We then assessed the long-term reliability of the complete reagent decontamination procedure applied to the treatment of the home-made reaction mix to ensure complete elimination of contaminants. Compatible reagents were UV-irradiated while others (*Taq*, primers and dNTP) were treated with hl-dsDNase and reagents were then combined. Only SYBR Green I was not treated. We call the complete procedure UVD-decontamination and it is described in detail in the Materials and Methods section and in the flow chart in Figure 4. We used this procedure to analyse both ancient bovine DNA in a number of Pleistocene and Holocene archaeological bovine bone samples. Out of 409 NTCs using bovine-specific primers BB3/4 amplifying a 153 bp fragment and 279 NTCs testing amplicons between 79 and 94 bp in length (for details see Methods S1), not a single one yielded a PCR product despite the high cycle number of 60 cycles that we routinely use. As can be seen on Table S2, with such a low level of contamination reliable results even from samples with poor DNA preservation can now be obtained. For example, when 50 samples are analyzed and multiple comparisons are corrected with the Bonferroni procedure, samples can be validated when duplicated through at most 9 or 14 attempts, depending on the fragment size considered. Thus, when using a home-made mix with individually tested clean reagents, in combination with the proposed UVD-decontamination procedure, even low level contamination with very small DNA fragments can be efficiently removed, and very sensitive PCR amplifications can be reliably conducted.

The complementation of the enzymatic treatment with irradiation is necessary due to the incompatibility of the optimal concentrations of the buffer compounds for maximal activity of the dsDNase and the *Taq* polymerase. In particular, for subsequent use in PCR, PCR reagents require a DNase treatment of highly concentrated stock solutions (such as Tris, detergent, MgCl_2_, KCl), which is incompatible with the activity of the dsDNase [82]). To guarantee maximal decontamination of these stock solutions, quantities of dsDNase would be needed that we showed to inhibit the *Taq* polymerase (1 U and 2 U dsDNase decreased the efficiency of the PCR by 20%, 4 U by 40% compared to untreated *Taq* polymerase). In addition, direct treatment of the PCR mix with hl-dsDNase prior to the addition of exogenous DNA was unsatisfactory because the experimental conditions allowing optimal activity of the hl-dsDNase were found to adversely affect the sensitivity of the PCR by a factor of roughly 10. The convenient solution to this problem is presented here with the UVD decontamination procedure combining UV and DNase treatment.

Depending on the contamination source it is probably not necessary to treat all reagents, e.g., in the case of contamination with DNA from domestic animals. When working with target molecules that are ubiquitously present in the environment, however, such as human and bacterial DNA, the complete UVD-decontamination procedure should be applied.

We also analyzed the influence of the UVD treatment on the fidelity of the *Taq* polymerase. Indeed, since optimal hl-dsDNase treatment requires extra Mg^2+^ and Ca^2+^ ions, the presence of these ions in the final PCR could have affected its efficiency and fidelity. Therefore, we amplified five independent target sequences between 73 and 358 bp with and without UVD treatment. We observed no significant decrease in amplification efficiency. The 78 PCR products obtained were verified by sequencing and found to be identical. Thus, there was no indication that the treatment with hl-dsDNase has an effect on the fidelity of the *Taq* polymerase.

### Conclusion

Identification of contamination sources and prevention of contamination constitute a key strategic challenge when attempting PCR amplification of minute quantities of DNA, and can have a major impact on the quality and reliability of the data produced. Contamination sources are multiple and diverse and contamination levels can fluctuate considerably, e.g., carry-over contamination levels vary depending on the previous amplification history and high lot-to-lot variability in reagent contamination levels can be observed. Moreover, contamination prevention methods have varying efficiency and these efficiencies also fluctuate, e.g., UV- and γ-irradiation efficiencies can be influenced by the spatial distribution of the contaminated source with respect to the irradiating device and the activity of degrading enzymes show lot-to-lot variations. These fluctuations make contamination prevention difficult and require extensive controls to accurately determine the true extent of contamination. Since individual contamination prevention strategies vary in efficiency, and contamination sources also fluctuate, a robust contamination prevention procedure should use multiple redundant strategies. Indeed, it is probably unrealistic to expect that a single procedure will be sufficient in all situations. Finally, even when maximal caution is exerted during the analysis of trace amounts of DNA, it is essential to systematically perform a large number of control amplifications. The number of total NTCs required depends on the success rate of the sample amplifications and can be estimated using a statistical test. With poorly preserved samples, several hundreds of control amplification may be required. Reagent decontamination prior to the analysis decreases the contamination background noise and thus the probability of erroneous results, of which the authenticity cannot be ascertained unless extensively reproduced.

In summary, using a quantitative approach, we tested a large number of previously described decontamination agents and developed a decontamination procedure that can be used for highly sensitive and efficient PCR systems. Importantly, we tested decontamination using a sensitive qPCR approach targeting small DNA fragments (as small as 73 bp), which allows analysis of highly degraded samples. To remove most of the contaminating DNA contained in PCR reagents, we propose UV-irradiation in a UV-crosslinking device for 10 minutes at shortest distance possible to the UV source. This procedure can be easily and routinely applied to batches of reagents, which can then be stored at −20°C for later use. Primers, dNTPs and the *Taq* polymerase, however, cannot be UV-irradiated and require another decontamination treatment using a heat-labile ds-specific DNase. Our results show that UV-irradiation of the PCR buffer for 10 minutes removes 99–99.9% of possible contaminants. Hl-dsDNase® destroys 99–99.9% of the contamination although the treatment of the *Taq* DNA polymerase allows merely a 99% decontamination level when performed in conditions that ensure nuclease inactivation without affecting significantly the *Taq* polymerase. Thus, this easily applicable protocol ensures that at least 99% of any kind of contaminating DNA molecules contained in the PCR reagents are degraded. Since reagents are mostly contaminated with small quantities of exogenous DNA, this should reduce the probability of contamination by the reagents to a negligible minimum.

We conclude that the use of the UVD-decontamination procedure significantly reduces the number of false positives due to contaminating DNA of human or animal origin in PCR reagents. It greatly improves the reliability of the results obtained from samples containing minute quantities of target DNA when it is coupled with enzymatic carry-over decontamination, physical containment and strict experimental procedures and the use of bleach to decontaminate working surfaces and equipment. Thus, the procedure proves to be an important progress in the areas of forensic genetics, wildlife and ancient DNA research of species that are prone to contaminate reagents, i.e., humans and some domestic animals such as pigs, cows, and presumably also in pathogen detection.

Although high throughput sequence analysis using the most recent next generation sequencing methods (e.g., [8], [84]) are believed to be less prone to contamination-borne errors, they are nevertheless sensitive to contamination during the first experimental steps where trace amounts of DNA molecules are treated with enzymes to allow enrichment and amplification (e.g., [85]; [86]). Thus, our decontamination method may prove useful for ultrasensitive next-generation sequencing applications as well. Furthermore, standard PCR is by far less costly than next-generation sequencing and will remain useful for many studies that do not require a large amount of sequence information and for which costs is a concern as in clinical routine tests, conservation biology and for a large proportion of archaeological researches using ancient DNA analyses.

## Materials and Methods

Details of the experimental protocols used can be found in the Methods S1.

### PCR Amplification system

To ensure elimination of carry-over contamination, we relied on the UQPCR method replacing dTTP by dUTP and using uracil-N-glycosylase (UNG) treatment prior to each quantitative real-time PCR amplification [12]. The qPCR experiments were carried out for 60 cycles in the most sensitive qPCR format, the Lightcycler 2.0 apparatus (Roche Applied Science, Mannheim, Germany). We either used commercial Master mixes for qPCR, i.e., PLATINUM® Quantitative PCR SUPERMIX-UDG (Invitrogen, St. Louis, MO, USA) and FastStart DNA Master^PLUS^ SYBR Green mix (Roche Applied Science, Mannheim, Germany), or a home-made qPCR mix prepared according to Lutfalla and Uze [46]. Using the home-made qPCR mix, various *Taq* polymerases were tested for contamination with exogenous DNA: AmpliTaq Gold and AmpliTaq 360 DNA Polymerase (Applied Biosystems, CA, USA), FastStart Taq DNA polymerase (Roche Applied Science, Mannheim, Germany), HotStarTaq® DNA polymerase (Qiagen, Düsseldorf, Germany), GoTaq® Hot Start Polymerase (Promega Corporation, Madison, WI, USA).

Contamination of PCR reagents with foreign DNA was tested using primers amplifying the *B. taurus* and the human mitochondrial D-loop. The development of DNA decontamination methods was performed with DNA fragments used only for this purpose. To test the efficiency of various treatments designed to minimize contamination spread by the experimenter, we amplified a 107 bp DNA fragment of the ribosomal protein 49 (*rp49* gene) of *D. melanogaster*. To measure the efficiency of the irradiation and endonuclease treatments to degrade fragments of different sizes, we used phage λ DNA as a PCR template with PCR primers amplifying regions of various lengths from 73 bp to 307 bp (including the primer annealing sites). To examine the DNase I treatment, we amplified a 103 bp fragment of the tetracycline resistance gene of plasmid pBR322 (see Table S1).

### Decontamination treatments of surfaces and equipment

We tested the efficiency of six decontamination treatments designed to prevent the spread of exogenous DNA that might be deposited on surfaces and transferred by latex gloves. The treatments tested are currently in use in the forensic and ancient DNA field: (1) UV light using a calibrated manual UV lamp, (2) DNA away®, (3) “CoPA solution” (copper-bis-(phenanthroline)-sulfate/H_2_0_2_ solution; US patent n° 5858650), (4) bleach, (5) soap and (6) air showering (http://www.chem.ox.ac.uk/OxfordTour/abc/node9.html). These tests were carried out on serial dilutions of target DNA that were applied to latex gloves, treated with the various treatments and amplified (see Methods S1). From the qPCR amplifications, we determined the degree of DNA degradation and the influence on PCR efficiency induced by the decontamination treatments compared to an untreated control and a control that had been treated with water (water control).

### Irradiation

γ-Irradiation experiments were carried out on serial dilutions of λ-DNA with a ^137^Cs source that had been calibrated by Fricke dosimetry. The efficiency of γ-irradiation to destroy double-stranded DNA was assayed by amplifying DNA fragments of various sizes from 73 bp to 307 bp. The effect on PCR performance of irradiation of the qPCR reaction buffer, of the FastStart Taq DNA polymerase and of the PCR primers was assayed by performing qPCR experiments on non-irradiated phage λ DNA with PCR mixes prepared with separately irradiated buffer components and reagents and compared with a control reaction performed with non-irradiated reagents.

UV decontamination of reagents was performed as described for the γ-irradiation experiments. Serial dilutions of λ-DNA were UV-irradiated in thin-wall, UV-clear tubes (Abgene, Epsom, UK or Invitrogen, Carlsbad, USA) for 10 minutes using a Stratalinker® 2400 device (Stratagene, Cedar Creek, USA) or a Spectrolinker XL 1500 UV crosslinker device (Spectronics Corp. Westbury, NY, USA). The extent of decontamination was then evaluated using qPCR. Similarly, the efficiency of PCR amplification achieved with each individually irradiated reagent was measured.

### Endonuclease Treatment

We tested whether DNase I, a nuclease that cleaves single- and double-stranded DNA without sequence specificity, could be used as a decontamination agent for PCR reagent's contamination. In particular, we tested the double-strand specificity of DNase I (Roche Applied Science, Mannheim, Germany) and whether primers with the tendency to form secondary structures could be a substrate for DNase I.

We also tested the efficiency of double strand-specific endonucleases from arctic shrimp including a heat-labile mutant version (dsDNase and hl-dsDNase, respectively; Biotec Marine Biochemicals, Tromsø, Norway; [82]), to degrade DNA molecules of various molecular weights. For these experiments, we treated phage λ DNA with each of the dsDNases and assayed the level of DNA degradation by amplifying regions of various lengths from 73 bp to 307 bp using qPCR as described above. We also adapted the buffer conditions of this enzyme to ensure the maximum reduction of contaminating bovine and human DNA in *Taq* polymerases and primers without reducing PCR sensitivity and efficiency.

### Recommended decontamination procedure

We recommend the following protocol to decontaminate PCR reagents prior to PCR amplification (see Figure 4).


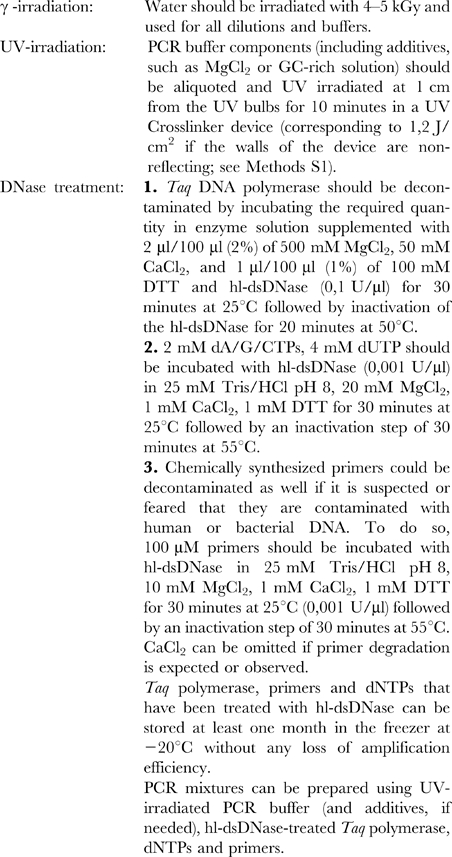


Supporting information is also available as a single pdf file for printing or offline reading (Fig. S5).

## Supporting Information

Methods S1 (0.09 MB DOC)

Figure S1 Relationships between the numbers of NTCs performed and the PCR contamination level that can be excluded with a 95% confidence level. The exact binomial test [11] was used to calculate the one tailed probability that a certain number of NTCs performed is significantly lower than a certain theoretical proportion of contaminated PCR reactions. The simulation was performed for various proportions when 0, 1 or 2 NTCs gave an amplification product (red, blue and green lines, respectively), and the minimal number of NTCs that gave a probability of 0.05 or lower was plotted. The curves can be used to determine the lowest percentage of contamination that can be excluded as a function of the number of NTCs performed. For best precision, two versions of the curves are shown with different scales in the abscissa and the ordinate. For example, if 100 NTCs have been performed and only one yielded a positive PCR, then one can assume with a 95% confidence level that the absolute contamination level of the PCRs is lower than 4.7%, whereas if only 30 NTCs are performed, even without yielding a single positive PCR, then one can only assume that the contamination level is below 9.5%.
(0.17 MB TIF)

Figure S2 Genetic diversity of mitochondrial sequences contaminating PCR reagents. Median Joining networks [8] of products from negative PCR controls (NTCs) using primer pairs BB3/4 and BB1/2. A. Bos taurus sequences. The sequence distribution observed in the contaminant resembles that of the European bovine mitochondrial sequences: the bovine sequences show a predominance of the T3 haplogroup with a few closely related sequences [12]. B. Sus scrofa. The porcine sequences are more diverse and include, at a lower resolution, haplogroups described by [13]: a is contained in haplogroups EH1, 2, 3, 7, 9, 10, 11, 13, 14, 18, 19, 22, 23, 24, 25; b is contained in haplogroups EH16 and 20; c is contained in EAH3, 4 and AH18, 24 and 27; d is contained in haplogroup EAH1; e is contained in haplogroups EAH2 and AH19. Haplogroups starting with letter E correspond to European breeds, whereas haplogroups starting with letter A correspond to Chinese breeds.
(0.05 MB TIF)

Figure S3 DNase I treatment. A. Effects of the DNase I treatment on the degradation of primers. Radio-labeled primers BR1 and BR2 had been incubated with DNase I for 10 minutes at 37°C at a concentration allowing complete degradation of a 103 bp fragment of plasmid pBR322, followed by 10 minutes of heat inactivation at 95°C prior to gel electrophoresis in a 12% polyacrylamide gel followed by autoradiography. Most of primer BR1 was degraded whereas primer BR2 was essentially unaffected. Primer BR1 was predicted to form a hairpin structure stable at 37°C, which is represented below. B. Efficiency of PCR after treatment of DNase I-sensitive primers. Primers (BR1 and BR2) were treated with DNase I in PCR reaction buffer in the absence of Taq followed by heat inactivation at 95°C for 10 minutes. Since the heat inactivation step lead to bovine serum albumin (BSA) precipitation, the reaction was centrifuged and fresh BSA and hot start Taq was added. QPCR was then performed on titration series of pBR322. Although DNase I treatment did not lead to a delay of the PCRs, the reaction plateau was reached much earlier and thus less DNA was synthesized. This effect is attributed to primer degradation. Green: control PCR; blue: DNase I-treated reaction mix. C. Hot start DNA polymerase is prematurely activated by a “mild” heat inactivation step that allows DNAse I inactivation. A horse DNA titration curve ranging from 100 to 0.16 pg, alongside 5 non-template controls (NTC), were amplified using primer pair EA51-61 (83 bp) in the Roche FastStart DNA MasterPLUS SYBR Green I mix supplemented with 30 mM DTT. PCR reactions were either incubated at 80°C for 30 minutes or not prior to PCR amplification with the following protocol: 15 min. at 37°C (UNG incubation step); 5 min. at 95°C (hot start activation step); followed by 60 cycles of 5 sec. at 95°C, 40 sec. at 65°C. Dimers were identified by a subsequent melting curve analysis. The figure represents the Ct of each sample as a function of the log of the concentration of DNA. The heat-treated samples are represented by blue diamonds, whereas the control samples are represented by red triangles.
(0.58 MB TIF)

Figure S4 hl-dsDNase treatment of primers. PCR efficiency after treatment of DNase I-sensitive primers BR1/2 with hl-dsDNase. Blue diamonds  =  Control: PCR with untreated primers BR1/2; red squares =  PCR with primers BR1/2 that were treated separately; green triangles  =  PCR with primers BR1/2 treated together.
(0.05 MB TIF)

Figure S5 Supporting information as a single pdf file for printing or offline reading.
(0.82 MB PDF)

Table S1 Primers used for amplication of various target molecules.
(0.02 MB DOCX)

Table S2 Examples of significantly different distributions between sample and control PCRs. When sample and contaminating sequences are indistinguishable, to ensure authenticity of the sample amplification with a 95% confidence level it is necessary to demonstrate that the rate of success of sample amplification is significantly higher than the background of contamination detected in the PCR blank controls (NTCs). The table presents various examples of threshold values that are different with a P-level of significance (α) of 0.05 or lower as determined using the Fisher's exact test. The left column with the blue background contains examples of various total numbers of NTCs performed and numbers of contaminated PCRs obtained (Positive NTCs/Total NTCs) that were chosen to either show cases (i) where only a small number of NTCs is carried out, even without obtaining positive amplification (from 0/2 to 0/100), or (ii) that were observed in the present study (from 0/279 to 151/1170), or (iii) that were chosen to present hypothetical situations where a very high number of NTCs was performed allowing detection of a few contaminated amplifications (from 50/1000 to 5/1000). For each of these NTCs values, we determined the threshold values of the minimal number of successful sample replications that must be obtained following a maximal number of PCR attempts (Positive Sample PCRs/Total Sample PCRs) to ensure that the chance that all sample amplifications are due to contamination is no more than 5%. The P-value will be lower than 0.05 when more replications are successful or when successful replications are obtained with fewer PCR attempts. When the rate of PCR success required is very high, this minimal number could prevent the validation of sequences obtained from samples that do not show very good DNA preservation and do not yield a product with every PCR attempt. Thus, the table also indicates for these cases the alternative replication number just above this minimal number, which would allow validation of sequences obtained from such less well-preserved samples. Finally, when several samples are compared to a single series of NTCs, there is an increased probability that an apparently significant difference can be obtained by chance and it is necessary to correct for multiple comparisons. This correction was performed using the conservative Bonferroni correction where the P-value α is adjusted by the number n of samples analyzed using α/n [11]. The table presents the numbers required to validate any sample using three examples of multiple comparisons: either when analyzing a single sample (no correction), or 10 or 50 samples.
(0.05 MB DOC)
