# Supplementary material for: An Efficient Multistrategy DNA Decontamination Procedure of PCR Reagents for Hypersensitive PCR Applications
Source: PLoS One. 2010 Sep 28;5(9):e13042. doi: 10.1371/journal.pone.0013042 (PMC2946917; doi:10.1371/journal.pone.0013042)
Supplement: Methods S1 — (0.09 MB DOC) [file pone.0013042.s001.doc]

Supporting information for:

**An Efficient Multistrategy DNA Decontamination Procedure of PCR reagents for Hypersensitive PCR Applications**

Sophie Champlot ^§^, Camille Berthelot^+§^, Mélanie Pruvost*, E. Andrew Bennett, Thierry Grange and Eva-Maria Geigl

Institut Jacques Monod, UMR7592 CNRS Université Paris 7, Paris, France

*present address : Deutsches Archäologisches Institut, Berlin, Germany

^+^present address: Institut de Biologie de l'Ecole Ecole Normale Supérieure, S2 Génomique Fonctionnelle - CNRS UMR 8197, INSERM U1024, Paris, France

^§^These authors contributed equally to this work

**Supporting Information and Methods**

**1. Ancient DNA facility**

PCRs for the detection of reagent’s contamination with animal, human and bacterial DNA were prepared in PCR flow hoods equipped with UV light and Biozone^TM^ AirCare^TM^ devices in a high containment facility with positive air pressure (http://www.ijm.fr/ijm/plates-formes/pole-paleogenomique-et-taphonomie-moleculaire/) using highly stringent experimental procedures as described .

**2. qPCR Amplification system**

PCR amplifications were performed using the UNG-coupled quantitative real-time PCR system UQPCR . QPCR reaction buffer (10 x) was prepared as described by Lutfalla and Uze , except for a total replacement of dTTP by dUTP, and addition of *Taq DNA polymerase (*FastStart Taq DNA polymerase, Roche Applied Science, Mannheim, Germany) prior to PCR rather than in the 10 x qPCR buffer. dNTPs were from Roche Applied Science, (Mannheim, Germany) since the latter are certified to originate from chemical synthesis. In contrast, other manufacturers confirmed to produce parts of their dNTPs from animal products (e.g., InVitrogen, pers. comm.), which therefore bear a high potential of being contaminated with DNA from domestic animals. The final composition of the 1 x qPCR reaction buffer is 1.6 % glycerol, 40 mM 2-amino pH 8.3, 5 mM KCl, 50 µg/ml horse serum albumin HSA (Sigma A 3434; Sigma-Aldrich Inc., St. Louis, MO, USA), 0.024 % Lubrol (2-dodecoxyethanol;Sigma-Aldrich). The qPCR conditions used with primers BB3/4 were 1 U FastStart Taq (Roche Applied Science), 1 x qPCR buffer, 2 mM MgCl_2_, 1 x GC-rich solution (Roche Applied Science), 1 µM of each primer, 30µM dA/G/CTPs and 60 µM dUTP. The qPCR experiments were carried out in a Lightcycler 2.0 apparatus (Roche Applied Science Mannheim, Germany) with the following program: a 10 minute-activation step at 95°C was followed by 60 cycles of denaturation (95°C, 15s), annealing (56°C, 15s) and elongation (67°C, 20s), followed by one melting step (95°C for 15s, then 60°C for 10s and temperature increase of 0.1°C per 10s with continuous fluorescence measurement) when the melting temperature Tm of the amplification products were measured. These conditions varied according to the primers (e.g., GC-rich solution was used only for primers BB3/4 and BB1/2). All PCRs were amplified for 60 cycles.

Decontamination tests were carried out on various DNA species and primer sequences are given in Table S1. To test the degradation of DNA by irradiation, we used phage λ DNA as a PCR template, with PCR primers amplifying regions whose lengths (including the primer annealing sites) range from 73 bp (L9/L5), 104 bp (L1/L3), 150 bp (L1/L4), 153 bp (L9/L10), 188 bp (L1/L5), 244 bp (L1/L6) to 307 bp (L1/L7)*.*

To amplify contaminating bovine DNA from commercial reagents, we used PLATINUM® Quantitative PCR SUPERMIX-UDG (Invitrogen, St. Louis, MO, USA) and FastStart Taq DNA polymerase (Roche Applied Science, Mannheim, Germany), and primers that had been designed to amplify the D-loop region of bovine mitochondrial DNA: BB1 and BB2 amplify a 201 bp fragment (including the primer annealing sites) and cover nucleotide positions 16,160-16,361 of the taurine mitochondrial consensus sequence according to Anderson ; BB3 and BB4 amplify a 153 bp fragment (including the primer annealing sites) and cover nucleotide positions 16,022-16,175. The contamination of LightCycler® FastStart DNA Master^PLUS^ SYBR Green I mix (Roche Applied Science, Mannheim, Germany) was assayed likewise using a primer pair targeting a 1107 bp fragment of the mitochondrial D-loop region between nucleotide positions 15,623 and 390 with primers BOUT2 and BOUT3, a 702 bp fragment with a combination of primers BB3 and BOUT3, and a 555 bp fragment with primers BOUT2 and BB4 and the aforementioned primers BB3 and BB4 (Table S1).

To test for the efficiency of the DNase I treatment, we amplified a 103 bp fragment of the *tet* sequence of plasmid pBR322 using PLATINUM® Quantitative PCR SUPERMIX-UDG and primers BR1 and BR2 (Table S1).

To test for the efficiency of various treatments designed to minimize spread of contamination via contact of the experimenter, a 107 bp fragment of the ribosomal protein 49 (*rp49* gene) of *D. melanogaster* was amplified in the presence of dUTP from genomic DNA of *D. melanogaster* using the FastStart DNA Master^PLUS^ SYBR Green mix (Roche Applied Science, Mannheim, Germany) and primers RPF and RPR.

Various *Taq* polymerases were tested for contamination with bovine DNA using primers BB3/4 and the decontaminated home-made PCR buffer as described above, according to the manufacturer’s recommendations: AmpliTaq Gold® (Applied Biosystems, CA, USA), FastStart Taq DNA polymerase (Roche Applied Science, Mannheim, Germany), HotStarTaq® DNA polymerase (Qiagen, Hilden, Germany), GoTaq® Hot Start Polymerase (Promega Corporation, Madison, WI, USA).

Identification and characterization of the PCR products were performed by analysis of the fusion temperature Tm of the products using the Lightcycler® and when necessary, by electrophoresis of 1 µL of the qPCR mix in a 10 % polyacrylamide gel.

The efficiency of the PCR was evaluated by determining the slope of the regression line obtained when plotting the Cts (crossing points at threshold) as a function of the logarithm of the concentration of the relative initial template quantity. The amplification efficiency was deduced from the slope of the linear regression analysis using the formula 1+E=10^-1/slope^ .

The efficiency of DNA degradation (in %) was calculated using the following equation :

$$\text{Edeg}=\frac{100}{\text{(1}+\text{E)}^{\text{ΔCt}} }$$

where E is the efficiency of the PCR amplification varying between 0 and 1 as deduced from the slope of a standard curve, and ΔCt is the difference between the Ct of treated and control samples.

The test of the efficiency of the UNG treatment was performed according to Pruvost et al. using amplicons of the tetracycline resistance gene of pBR322 in which thymines had been replaced by uracil. The activity of the UNG from a marine bacterium (Roche Applied Science, Mannheim, Germany) and the UNG extracted from cod (*G. morhua*; Biotec Marine Biochemicals, Norway) was compared by quantifying the DNA before and after treatment using qPCR.

**3. Decontamination treatment of surfaces and equipment**

To test for the efficiency of decontamination procedures of surfaces, six decontamination agents were tested: (1) UV light, (2) DNA away®, (3) “CoPA solution”, (4) bleach, (5) detergent and (6) air showering.

As a source for UV light we either used a UV Stratalinker (Stratalinker® UV Crosslinker 2400 device Stratagene) or a calibrated manual UV lamp.

According to US patent n° 5858650, a 10 mM copper-bis-(phenanthroline)-sulfate solution was prepared by dissolving 10 mM anhydrous copper sulfate and 20 mM 1,10-phenanthroline hydrate by sonication in water. The 10 mM copper-bis-(phenanthroline)-sulfate solution was mixed with two volumes of 6.8 % H_2_0_2_ to yield the “CoPA solution” used to wipe working surfaces and equipment.

We tested for the efficiency of various decontamination treatments currently in use in the forensic and ancient DNA field to prevent dispersal of exogenous DNA attached to gloves and the skin of the experimenters. The 107 bp *rp49* gene of *D. melanogaster* was amplified from genomic DNA. The amplification product was quantified in a nanodrop device (NanoDrop^TM^ 2000, Thermo Fisher Scientific Inc., Wilmington, DE, USA) and serially diluted. Squares of 1 cm^2^ were cut from powderless latex gloves. 10 µl of the serial dilutions of the *rp49* amplicon (between 170 pg and 17 fg) were applied to each square and air-dried. For the negative controls, 10 µl of water was applied. The squares containing different quantities of DNA were then treated in different conditions as followed: (i) Some were exposed for 1 hour at a distance of 10 cm to a manual UV lamp that delivered at this distance an energy of 4 J/m^2^, thus corresponding to a total delivery of 1.45 J/cm^2^. Other squares were rubbed with a cotton swab soaked in either (ii) water (negative controls), (iii) “CoPA solution”, (iv) Bleach (“Eau de Javel”, La Croix, containing 2.6 % active chlorine), (v) DNA away® (Molecular Bioproducts, San Diego, CA, USA) and (vi) detergent (a 10% dilution of dish-washing detergent containing 5-15% anionic non-ionic surfactants). To test recommended air showering procedure (<http://www.chem.ox.ac.uk/OxfordTour/abc/node9.html>), air in a syringe was pressed through a filter on a glove square containing dried DNA. To recover the DNA the glove squares were then incubated over-night in a microtube at 37°C in 200 µl TE buffer (10 mM Tris/HCl pH 8; 1 mM EDTA). Using a heated needle, a hole was introduced in the bottom of the microtube while it was placed over a fresh microtube. After centrifugation in an Eppendorf centrifuge at maximum speed for 1 minute, DNA was ethanol-precipitated using 20 µl 0.25% linear polyacrylamide as a carrier , washed, dried, dissolved in water and amplified by qPCR using primers PRF/R.

**4. Reagent’s contamination**

Negative PCR controls (NTCs) with primer pairs BB3/4 or BB1/2 that yielded a product were sequenced. The sequences obtained were analysed via the median joining network approach and are shown in Figure S1.

**5. Irradiation**

*5.1. UV-irradiation*

UV-irradiation tests were carried out in a Stratalinker® UV Crosslinker 2400 device (Stratagene, Cedar Creek, USA) and a Spectrolinker XL 1500 UV crosslinker device (Spectronics Corp. Westbury, NY, USA), both operated by five 254 nm UV bulbs (15 W). With brand new UV bulbs, a 10 minute irradiation corresponds to an energy of 1.20 J/cm^2^ as measured by a cell at 11 cm from the bulbs when using an old generation Stratalinker® UV Crosslinker 2400 devices that have no reflecting walls. In the new generation Spectrolinker XL 1500 UV crosslinker devices with their reflecting aluminium walls, more UV light is received by the measuring cell and 10 minutes correspond to 4.8 J/cm^2^. Since reflecting walls do little to improve the light received by microtubes at 1 cm of the UV bulbs, the value of the energy received by the measuring cell is not accurately reflecting that received by the sample. To accommodate different generations of UV crosslinkers, we preferred to refer to the irradiation time instead, and to use the measurement of the energy received by the cell to monitor the decrease in performance due to aging of the UV bulbs. This value is then used to increase the irradiation time accordingly.

UV-irradiation was performed in thin-wall, UV-clear 0.2 ml PCR tubes (Abgene, Epsom, UK) for small volumes (10 µL – 200 µL) or in thin-wall, UV-clear 0.6 mL Qubit™ tubes (Invitrogen, Carlsbad, USA) for larger volumes, as other tubes gave poor results due to inefficient UV-light transmission. Tubes were irradiated lying on the side on an aluminium sheet at around 1 cm from the bulbs.

The efficiency of UV-rays to inactivate DNA for PCR was assayed by irradiating different volumes of bacteriophage λ DNA and performing qPCR as described above on a titration series (50 pg, 10 pg, 2 pg and 400 fg) of irradiated DNA to assess the extent of inactivation of the template, compared to a non-irradiated standard range. The primers used were L9-L5 targeting a 73 bp region (Table S1). Such experiments were carried out on phage λ DNA buffered either with water, 10 x qPCR buffer (without SYBR Green I dye), 25 mM MgCl_2_, or 5 x GC-rich solution (Roche Applied Science), in order to assess any UV-protective effect that could interfere with the decontamination process.

The effect of UV-rays on PCR performance of the qPCR buffer reagents and of the FastStart Taq DNA polymerase was assessed by carrying out PCR experiments using these irradiated reagents separately or together, and comparing the PCR efficiency to experiments performed with non-irradiated reagents. Phage λ DNA was used as a template and amplified using the L9-L5 primer couple as described above. Whenever 10 x qPCR buffer was irradiated, the SYBR Green I dye was added only after irradiation but before the –80°C freezing step , to ensure efficient DNA detection.

*5.2. γ-irradiation*

Irradiation experiments were carried out in an IBL 637 device with a ^137^Cs source that had been calibrated by Fricke dosimetry.

QPCR reaction buffer (10 x), FastStart Taq DNA polymerase (Roche Applied Science), bacteriophage λ DNA and λ-specific PCR primers were γ-irradiated with 1, 2 or 4 kGy. When relevant for the tests, different reagents (HSA, dNTPs, detergent Lubrol, KCl, 2-aminopropanediol, glycerol, water) were irradiated separately, and used to prepare buffers containing subsets or all of the irradiated reagents. QPCR reaction buffer (10 x), FastStart Taq DNA polymerase, bacteriophage λ DNA and λ-specific PCR primers were γ-irradiated with 1, 2 or 4 kGy.

The efficiency of γ-irradiation to destroy double-strand DNA was assayed by performing qPCR on a serial dilution (260 pg, 26 pg, 2.6 pg and 260 fg) of irradiated phage λ DNA as a PCR template, with PCR primers L9-L5, L1-L3, L1L4, L1-L5 and L1-L7 described above, compared to non-irradiated controls.

Degradation of DNA through γ- and UV-irradiation in various buffers was tested by irradiating 25 ng/µL of λ DNA with 4 kGy γ-irradiation and for 10 minutes UV irradiation in a UV crosslinker in water complemented with 2.5 M 2-aminopropanediol, 50 % glycerol, 10 mg/mL horse serum albumin (HSA) in water, 10 % Lubrol in water, or 20 mM dNTPs (dATP, dCTP, dGTP) and 40 mM dUTP, followed by amplification of a 73-bp DNA fragment.

**6. Endonuclease treatment**

*6.1. DNase I digestion of primers*

DNase I digestion was performed on primers BR1/2 amplifying a 107 bp fragment of plasmid pBR322. Radiolabeling of the primers was achieved using T4 polynucleotide kinase (New England Biolabs, Ipswich, MA, USA) and γ-^32^P-dATP. DNase I solution was prepared from DNase I powder (Roche Applied Science, Mannheim, Germany) at a concentration of 15 U/µl. Radiolabeled primer DNA was incubated at room temperature for 10 minutes, respectively, with 3 U of DNase I solution in the presence of 3 mM MgCl_2_ (Figure S2A.). In the example shown in Figure S2A, primer BR1 is highly sensitive to DNase I, which could be due to the formation of a hairpin and a very stable primer-dimer (ΔG = - 6,9 kcal/mol) that can be predicted by the software “Oligo6” (Molecular Biology Insights, Inc., Cascade, CO, USA) and that is shown below the gel picture in Figure S2A.

A 10 minute incubation of the PCR mix with DNase I at room temperature was sufficient to completely degrade any DNA present in the reaction mixture, as evaluated by gel electrophoresis analysis of a radiolabelled 103 bp pBR322 fragment that had been incubated with DNase I at different incubation times between 5 and 45 minutes (data not shown). The following heat-inactivation at 95°C for 10 minutes irreversibly denatured the DNase I prior to PCR as evaluated by gel electrophoresis of the same radiolabelled pBR322 fragment incubated with 3 U DNase I that had been heat-denatured between 5 and 20 minutes (data not shown). This denaturation treatment, however, prematurely activated the Hot-Start *Taq* polymerase. Consequently, the *Taq* DNA polymerase must be added after inactivation of the DNase I, which is not practical with large numbers of reactions and increases the risk of contamination. The compulsory heat denaturing of the DNase I also prematurely denatured the stabilizing proteins such as BSA leading to a largely delayed amplification (14 cycles on average) with a significantly decreased efficiency. When adding the albumin after DNase I treatment, the efficiency of the PCR was recovered but the saturation plateau was reached much earlier and only half of the quantity of DNA molecules were newly synthesized (Figure S2B).

We also tested whether a milder DNase I inactivation step could be performed without causing premature activation of the hot start DNA polymerase. Indeed, it was shown that DNAse I could be fully inactivated in the presence of 30 mM DTT by a heat treatment of 30 minutes at 80°C . We tested the effect of a 30-minute incubation at 80°C of the PCR reaction prior to perform the standard PCR protocol using primer pair EA51/61 amplifying 83 bp of horse mitochondrial DNA amplified using FastStart Taq DNA polymerase with a prior UNG treatment. Comparative analysis with a reaction mix containing the same concentration of DTT but that was not incubated for 30 minutes at 80°C reveals clearly that the Hot start DNA polymerase was significantly activated and primer dimers were appearing much earlier, thereby reducing the sensitivity of detection of low amounts of DNA (Fig. S2C). The reaction efficiency at the beginning of the PCR appears higher upon preincubation at 80°C, since the control DNA is detected earlier at the highest concentration, presumably because more active DNA polymerase is available in the initial phase of the PCR. However, dimers appear very early, in the experiment shown at least 15 cycles earlier than in the control reaction. Consequently, the low amounts of DNA cannot be measured because dimers are amplified earlier. Thus, this inactivation procedure is detrimental to the sensitivity of the PCR.

*6.2. Heat-labile dsDNase activity test*

To compare the activity of the hl-dsDNase® and the wild type dsDNase® (Biotec Marine Biochemicals, Tromsø, Norway), DNA degradation was measured by treating 100 pg of bacteriophage λ DNA with the dsDNases for 30 min at 25°C. DNase activity was tested in the following conditions. (i) In 25 mM Tris/HCl, pH 8, 10 mM or 20 mM MgCl_2_ and 1 mM CaCl_2_ in a final volume of 10 µl, with serial dilutions (with a
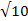
 serial dilution factor) of either dsDNase with concentrations ranging from 2 x 10^-3^ to 2 x 10^-5^ U/µl. (ii) In 20 µl FastStart Taq DNA polymerase storage buffer (20 mM Tris-HCl pH 9.0, 100 mM KCl, 0.1 mM EDTA, 1 mM DTT, 0.2 % Tween 20, 50 % glycerol) or in FastStart Taq DNA polymerase solution supplemented with 10 mM MgCl_2_, 1 mM CaCl_2_ and 1 mM DTT, were treated with 0.09, 0.01, 0.005, 0.0015 U/µl of dsDNase. The nuclease was inactivated by incubation for 30 minutes at 65°C.

QPCR was performed as described above on 1 µl of each of these assays in order to quantify the level of degradation of the λ DNA by the dsDNase. The extent of DNA degradation was assayed by performing qPCR on the treated DNA samples using the L9-L5 primer pair as previously described. Control qPCRs were performed on DNA samples where the DNase had been replaced by water.

*6.3. DNase inactivation test*

To test the efficiency of inactivation of both wild-type and hl-dsDNase (Biotec Marine Biochemicals, Tromsø, Norway), parallel experiments were performed with the two enzymes. Inactivation tests were carried out in different buffer conditions. (i) In 10 µl of 25 mM Tris/HCl, pH 8, 20 mM MgCl_2_ and 1 mM CaCl_2_, 1 U of nuclease was incubated for 10 or 30 minutes at 60°C or for 10 or 30 minutes at 55°C with or without 1 mM DTT. Residual activity was tested by an incubation step of the last mix with 100 pg of the bacteriophage λ in 250 mM Tris/HCl, pH8, 10 mM MgCl_2_ and 1 mM CaCl_2_ for 30 minutes at 25°C followed by a 30 minute-inactivation step at 65°C. (ii) In the storage buffer of the FastStart Taq DNA polymerase, within a final volume of 20 µl, 2 U of nuclease were incubated for 10 or 30 minutes at 55°C or for 10, 15, or 30 minutes at 50°C with or without 1 mM DTT in 10 mM MgCl_2_ and 1 mM CaCl_2_. Residual activity was tested by an incubation step of this mix with 100 pg of the bacteriophage λ in 250 mM Tris/HCl, pH 8, 10 mM MgCl_2_ and 1 mM CaCl_2_ for 30 minutes at 25°C followed by a 30 minute-inactivation step at 65°C. QPCR was performed as described above using the primers L9-5 and L9-10 with 1 µl of each of these assays to quantify to which degree the DNases degraded the λ DNA.

*6.4. Activity test of* dsDNases *on endogenous contaminations*

The efficiency of DNA degradation by the dsDNases on endogenous bovine contaminants co-purified with reagents was tested with the incubation of 1µl of the FastStart DNA Master^PLUS^ SYBR Green mix with or without 1 U of hl-dsDNase in a final volume of 10 µl of 25 mM Tris/HCl, pH 8 with 10 mM MgCl_2_, 1 mM CaCl_2_ and 1 mM DTT, for 30 minutes at 25°C and 30 minutes at 55°C. The treated and untreated Master^PLUS^ SYBR Green mixes were used for qPCR targeting the bovine mitochondrial control sequences with the primer pair BB3/4.

*6.5. Decontamination of Taq DNA polymerase, primers and dNTPs using hl-dsDNase*

The FastStart Taq DNA polymerase supplemented with 10 mM MgCl_2_, 1 mM CaCl_2_ and 1 mM DTT was incubated with hl-dsDNase (0,1 U/µl) for 30 minutes at 25°C followed by a 20 minute-inactivation at 50°C.

Primers were incubated with hl-dsDNase (0,001 U/µl) of nuclease for 800 pmol of primers) in 25 mM Tris/HCl, pH 8 buffer, 10 mM MgCl_2_, and 1 mM CaCl_2_, for 30 minutes at 25°C. The nuclease was inactivated for 30 minutes at 55°C.

dNTPs were incubated with hl-dsDNase (0,001 U/µl) for 2 mM of dATP, dCTP, dGTP and 4 mM of dUTP in 25 mM Tris/HCl pH 8 buffer, 1 mM CaCl_2_ and either 10 or 20 mM MgCl_2_, for 30 minutes at 25°C. The efficiency of decontamination was slightly better with 20 mM MgCl_2_, 98 % of the DNA was degraded whereas only 97.70 % with 10 mM MgCl_2_. The nuclease was inactivated for 30 minutes at 55°C.

The efficiency of the hl-dsDNase for decontamination of the *Taq* DNA polymerase, the primers and the dNTPs were assayed on a series of blank qPCR controls carried out with the primer pair BB3/4. The reagents, treated or untreated by DNAse, were tested in qPCR buffer where all other components but SYBR Green I were UV irradiated and where the volume of MgCl_2_ was adjusted so that the MgCl_2_ concentration provided by the nuclease-treated reagents was taken into account. A series of blank qPCR controls was carried out.

We also evaluated whether the *Taq* DNA polymerase and the primers could be treated together with hl-dsDNase. We found that under the conditions described before and at a MgCl_2_ concentration between 10 and 20 mM, 98 % of λ DNA was degraded when the treatment with hl-dsDNase was performed in the presence of 90 µM primers prior to PCR. Thus, the presence of primers does not interfere with the hl-dsDNase treatment. Subsequent PCR amplifications did not show a lower efficiency (difference in the efficiency ΔE=± 1%). Despite these results, separate treatment of the *Taq* DNA polymerase and of the PCR primers with hl-dsDNase is preferable if different primers are used. Furthermore, separate treatment minimizes the risk of primer-dimer formation during the inactivation step of the hl-dsDNase.

**7. Efficiency of the UVD decontamination procedure**

To assess the efficiency of the UVD decontamination procedure, we tested a large number of blank controls. In particular, no amplification products were obtained out of 409 NTCs using bovine-specific primers BB3/4 amplifying a 153 bp fragment, out of 164 NTCs using primers amplifying a 79 bp product, 70 NTCs using primers amplifying a 94 bp product from the bovine D-loop mitochondrial DNA, and 45 NTCs amplified with 7 different primer pairs of the bovine mitochondrial DNA amplifying products from 57 to 92 bp.

**8. Sequencing**

Sequencing was performed by capillary electrophoresis at Eurofins MWG Operon (Ebersberg, Germany) and by manual sequencing using the Sanger sequencing method and 5’-^32^P-phosphorylated sequencing primers .

**References**

1. Pruvost M, Schwarz R, Bessa Correia V, Champlot S, Braguier S, et al. (2007) Freshly excavated fossil bones are best for amplification of ancient DNA. Proc Natl Acad Sci USA 104: 739-744.

2. Pruvost M, Grange T, Geigl E-M (2005) Minimizing DNA contamination by using UNG-coupled quantitative real-time PCR on degraded DNA samples: application to ancient DNA studies. Biotechniques 38: 569-575.

3. Lutfalla G, Gilles U (2006) Performing Quantitative Reverse-Transcribed Polymerase Chain Reaction Experiments. Methods Enzymol 410: 386-400.

4. Anderson S, Bankier, A.T., Barrell, B.G. De Bruijn, M.H.L. Coulson, A.R., Drouin, J., Eperon, I.C., Nierlich, D.P., Roe, B.A., Sanger, F., Schreier, P.H., Smith, A.J.H., Staden, R., Young, G. (1981) Sequence and organization of the human mitochondrial genome. Nature 290: 457-465.

5. Stahlberg A, Aman P, Ridell B, Mostad P, Kubista M (2003) Quantitative real-time PCR method for detection of B-Lymphocyte monoclonality by comparison of k and l immunoglobulin light chain expression. Clin Chem 49: 51-59.

6. Thomassin H, Kress C, Grange T (2004 ) MethylQuant: a sensitive method for quantifying methylation of specific cytosines within the genome.

. Nucleic Acids Res 32: e168.

7. Gaillard C, Strauss F (1990) Ethanol precipitation of DNA with linear polyacrylamide as carrier. Nucleic Acids Res 18: 378.

8. Bandelt HJ, Forster P, Röhl A (1999) Median-joining networks for inferring intraspecific phylogenies. Mol Biol Evol 16: 37-48.

9. Silkie SS, Tolcher MP, Nelson KL (2008) Reagent decontamination to eliminate false-positives in Escherichia coli qPCR. J Microbiol Methods 72: 275-282.

10. Sanger F, Nicklen S, Coulson AR (1977) DNA sequencing with chain-terminating inhibitors. Proc Natl Acad Sci U S A 74: 5463-5467.

11. McDonald JH (2009) Handbook of Biological Statistics. Baltimore, Maryland: Sparky House Publishing.

12. Troy CS, MacHugh DE, Bailey JF, Magee DA, Loftus RT, et al. (2001) Genetic evidence for Near-Eastern origins of European cattle. Nature 410: 1088-1091.

13. Fang M, Andersson L (2006 ) Mitochondrial diversity in European and Chinese pigs is consistent with population expansions that occurred prior to domestication. Proc Biol Sci 273: 1803-1810.
