# Supplementary figures and images for: An Efficient Multistrategy DNA Decontamination Procedure of PCR Reagents for Hypersensitive PCR Applications
Source: PLoS One. 2010 Sep 28;5(9):e13042. doi: 10.1371/journal.pone.0013042 (PMC2946917; doi:10.1371/journal.pone.0013042)

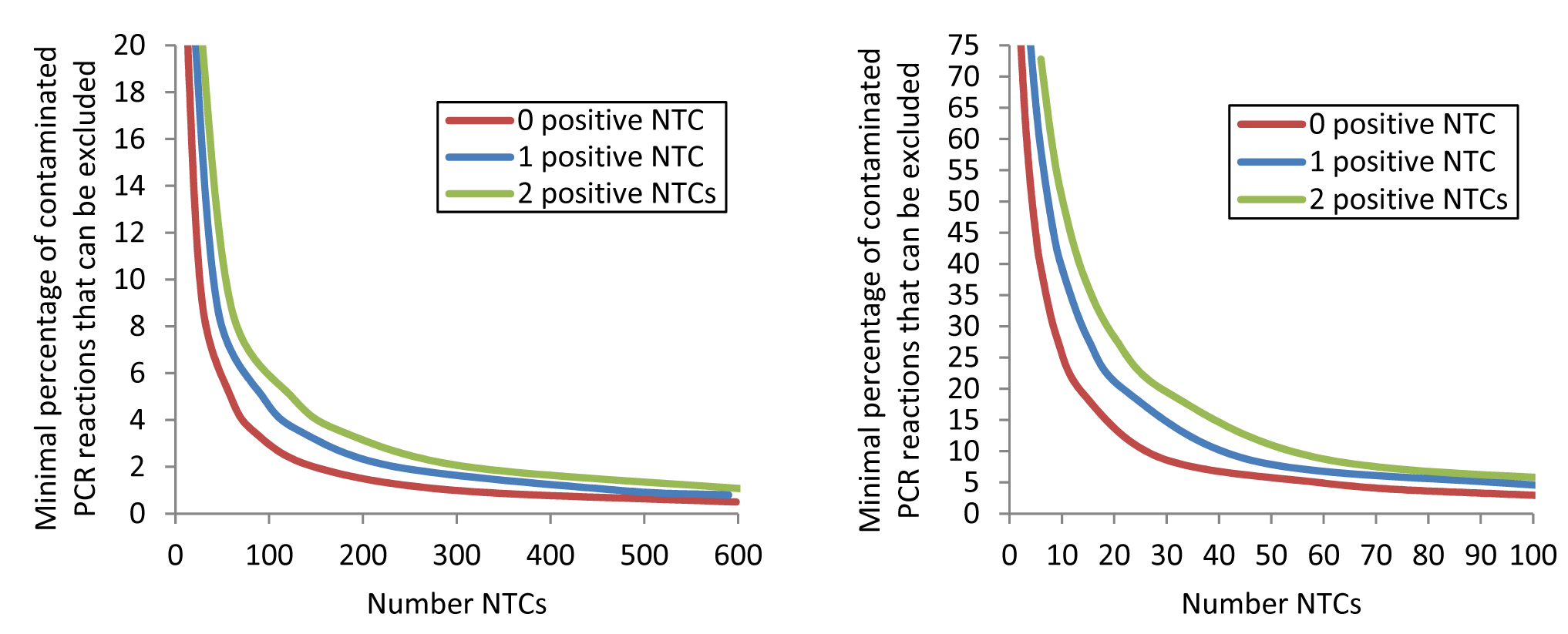

Supplement: Figure S1 — Relationships between the numbers of NTCs performed and the PCR contamination level that can be excluded with a 95% confidence level. The exact binomial test [11] was used to calculate the one tailed probability that a certain number of NTCs performed is significantly lower than a certain theoretical proportion of contaminated PCR reactions. The simulation was performed for various proportions when 0, 1 or 2 NTCs gave an amplification product (red, blue and green lines, respectively), and the minimal number of NTCs that gave a probability of 0.05 or lower was plotted. The curves can be used to determine the lowest percentage of contamination that can be excluded as a function of the number of NTCs performed. For best precision, two versions of the curves are shown with different scales in the abscissa and the ordinate. For example, if 100 NTCs have been performed and only one yielded a positive PCR, then one can assume with a 95% confidence level that the absolute contamination level of the PCRs is lower than 4.7%, whereas if only 30 NTCs are performed, even without yielding a single positive PCR, then one can only assume that the contamination level is below 9.5%. (0.17 MB TIF) [file pone.0013042.s002.tif]

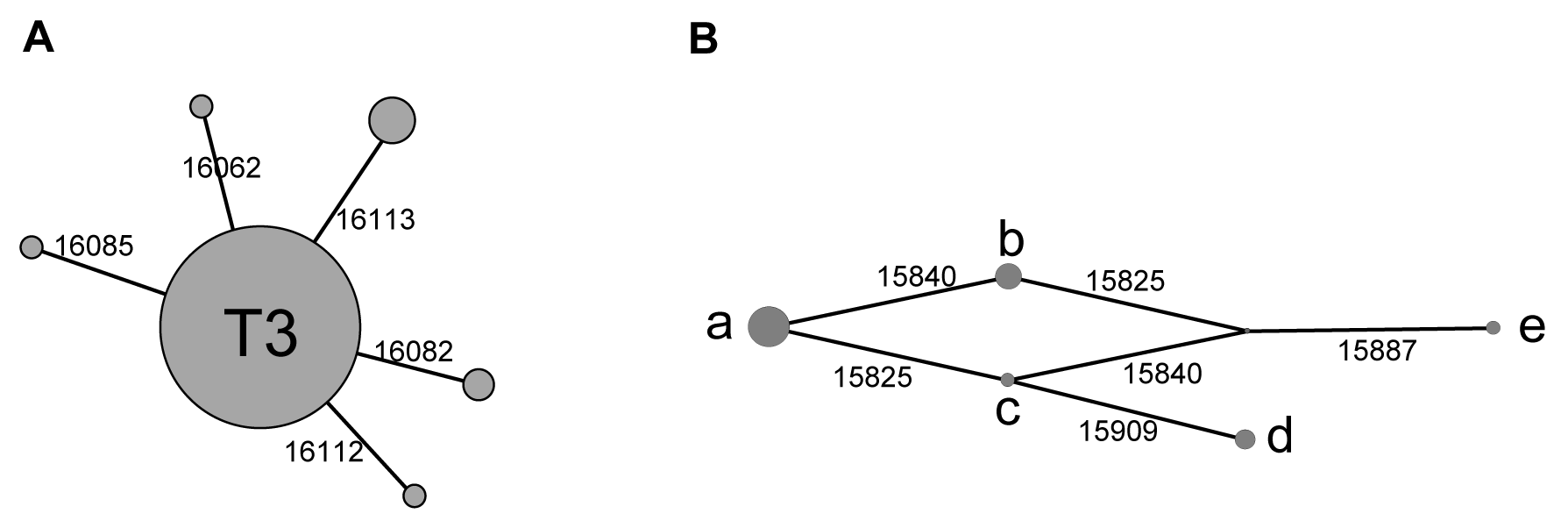

Supplement: Figure S2 — Genetic diversity of mitochondrial sequences contaminating PCR reagents. Median Joining networks [8] of products from negative PCR controls (NTCs) using primer pairs BB3/4 and BB1/2. A. Bos taurus sequences. The sequence distribution observed in the contaminant resembles that of the European bovine mitochondrial sequences: the bovine sequences show a predominance of the T3 haplogroup with a few closely related sequences [12]. B. Sus scrofa. The porcine sequences are more diverse and include, at a lower resolution, haplogroups described by [13]: a is contained in haplogroups EH1, 2, 3, 7, 9, 10, 11, 13, 14, 18, 19, 22, 23, 24, 25; b is contained in haplogroups EH16 and 20; c is contained in EAH3, 4 and AH18, 24 and 27; d is contained in haplogroup EAH1; e is contained in haplogroups EAH2 and AH19. Haplogroups starting with letter E correspond to European breeds, whereas haplogroups starting with letter A correspond to Chinese breeds. (0.05 MB TIF) [file pone.0013042.s003.tif]

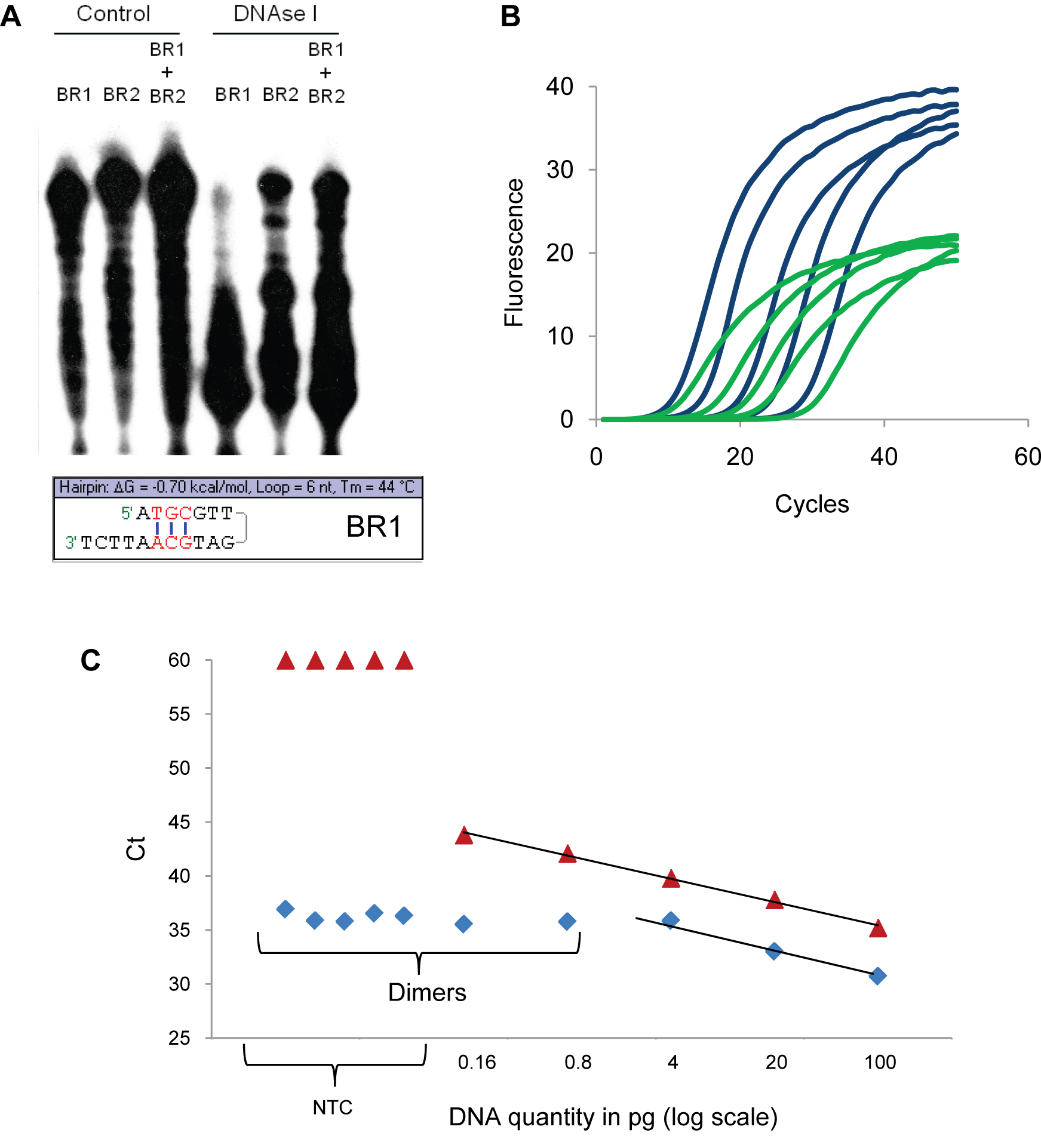

Supplement: Figure S3 — DNase I treatment. A. Effects of the DNase I treatment on the degradation of primers. Radio-labeled primers BR1 and BR2 had been incubated with DNase I for 10 minutes at 37°C at a concentration allowing complete degradation of a 103 bp fragment of plasmid pBR322, followed by 10 minutes of heat inactivation at 95°C prior to gel electrophoresis in a 12% polyacrylamide gel followed by autoradiography. Most of primer BR1 was degraded whereas primer BR2 was essentially unaffected. Primer BR1 was predicted to form a hairpin structure stable at 37°C, which is represented below. B. Efficiency of PCR after treatment of DNase I-sensitive primers. Primers (BR1 and BR2) were treated with DNase I in PCR reaction buffer in the absence of Taq followed by heat inactivation at 95°C for 10 minutes. Since the heat inactivation step lead to bovine serum albumin (BSA) precipitation, the reaction was centrifuged and fresh BSA and hot start Taq was added. QPCR was then performed on titration series of pBR322. Although DNase I treatment did not lead to a delay of the PCRs, the reaction plateau was reached much earlier and thus less DNA was synthesized. This effect is attributed to primer degradation. Green: control PCR; blue: DNase I-treated reaction mix. C. Hot start DNA polymerase is prematurely activated by a “mild” heat inactivation step that allows DNAse I inactivation. A horse DNA titration curve ranging from 100 to 0.16 pg, alongside 5 non-template controls (NTC), were amplified using primer pair EA51-61 (83 bp) in the Roche FastStart DNA MasterPLUS SYBR Green I mix supplemented with 30 mM DTT. PCR reactions were either incubated at 80°C for 30 minutes or not prior to PCR amplification with the following protocol: 15 min. at 37°C (UNG incubation step); 5 min. at 95°C (hot start activation step); followed by 60 cycles of 5 sec. at 95°C, 40 sec. at 65°C. Dimers were identified by a subsequent melting curve analysis. The figure represents the Ct of each sample as a function of the log [file pone.0013042.s004.tif]

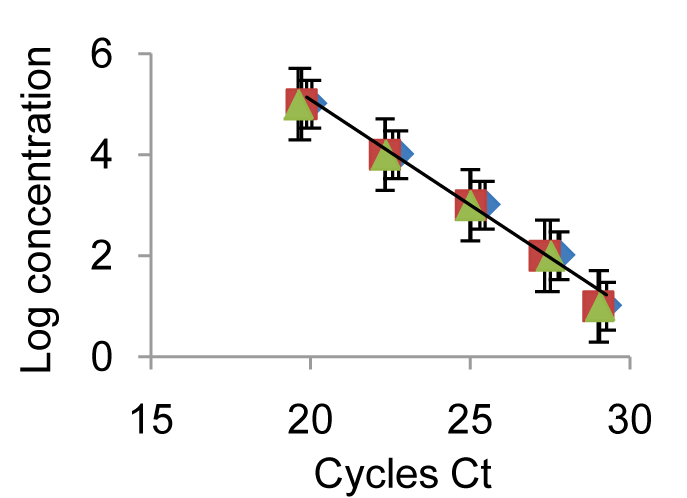

Supplement: Figure S4 — hl-dsDNase treatment of primers. PCR efficiency after treatment of DNase I-sensitive primers BR1/2 with hl-dsDNase. Blue diamonds = Control: PCR with untreated primers BR1/2; red squares = PCR with primers BR1/2 that were treated separately; green triangles = PCR with primers BR1/2 treated together. (0.05 MB TIF) [file pone.0013042.s005.tif]
