# Supplementary material for: An Efficient Multistrategy DNA Decontamination Procedure of PCR Reagents for Hypersensitive PCR Applications
Source: PLoS One. 2010 Sep 28;5(9):e13042. doi: 10.1371/journal.pone.0013042 (PMC2946917; doi:10.1371/journal.pone.0013042)
Supplement: Table S1 — Primers used for amplication of various target molecules. (0.02 MB DOCX) [file pone.0013042.s007.docx]

**Table S1: Primers used for amplication of various target molecules**.

| **target Sequence** | **primer name** | **primer sequence** |
| --- | --- | --- |
| phage λ | L1 | 5’-AAGGAAACGACAGGTGCTGA-3 |
| phage λ | L3 | 5’-CCAGCTGCTTTTTGTTGACTT-3 |
| phage λ | L4 | 5’-TCCTGCCAG TTCTGA ATGGT-3’ |
| phage λ | L5 | 5’-TACCCTTGCCACCGCCT-3’ |
| phage λ | L6 | 5’-AGCATCCCTTTCGGCATA-3’ |
| phage λ | L7 | 5’-ATCTGCCTCGCTGGCCT-3’ |
| phage λ | L9 | 5’-GGTGCGAGTATCCGTACCATT-3’ |
| phage λ | L10 | 5'-GCGCAGCTTTTCGTTCTC-3' |
| mitochondrial D-loop B. taurus | BB1 | 5’-TTAATTACCATGCCGCGTGA-3’ |
| mitochondrial D-loop *B. taurus* | BB2 | 5’-CATGGGCTGATTAGCCATTAGT-3’ |
| mitochondrial D-loop *B. taurus* | BB3 | 5’- CCATGCATATAAGCAAGTACATGA-3’ |
| mitochondrial D-loop *B. taurus* | BB4 | 5’-GCGGCATGGTAATTAAGCTC-3’ |
| mitochondrial D-loop *B. taurus* | BOUT2 | 5’-GGCACAATCGAAAACAAATTACT-3’ |
| mitochondrial D-loop *B. taurus* | BOUT3 | 5’-CTTGCTTTGGGTTAAGCTACATC-3’ |
| mitochondrial D-loop *B. taurus* | BB15m | 5'-TGA CTG TAC ATA GTA CAT TAT GTC AA-3' |
| mitochondrial D-loop *B. taurus* | BB16m | 5'-GCT CGT GAT CTA ATG GTA AGG-3' |
| mitochondrial D-loop *B. taurus* | BB17 | 5'-CCC CAT GCA TAT AAG CAA G-3' |
| mitochondrial D-loop *B. taurus* | BB18 | 5'-AAG AAT GAA TTT GAC ATA ATG TAC TA-3' |
| mitochondrial D-loop *E. caballus* | EA5.1 | 5'-CATCCAAGTCAAATCATTTCCA-3' |
| mitochondrial D-loop *E. caballus* | EA6.1 | 5'-CTGATTTCCCGCGGCTT-3' |
| pBR322 | BR1 | 5’-ATGCGTTGATGCAATTCT-3’ |
| pBR322 | BR2 | 5’-GTCGATAGTGGCTCCAAGTA-3’ |
| rp49 gene *D. melanogaster* | RPF | 5’-CCGCTTCAAGGGACAGTATCTG-3’ |
| rp49 gene *D. melanogaster* | RPR | 5’-CACGTTGTGCACCAGGAACTT-3’ |
