# Supplementary material for: An Efficient Multistrategy DNA Decontamination Procedure of PCR Reagents for Hypersensitive PCR Applications
Source: PLoS One. 2010 Sep 28;5(9):e13042. doi: 10.1371/journal.pone.0013042 (PMC2946917; doi:10.1371/journal.pone.0013042)
Supplement: Table S2 — Examples of significantly different distributions between sample and control PCRs. When sample and contaminating sequences are indistinguishable, to ensure authenticity of the sample amplification with a 95% confidence level it is necessary to demonstrate that the rate of success of sample amplification is significantly higher than the background of contamination detected in the PCR blank controls (NTCs). The table presents various examples of threshold values that are different with a P-level of significance (α) of 0.05 or lower as determined using the Fisher's exact test. The left column with the blue background contains examples of various total numbers of NTCs performed and numbers of contaminated PCRs obtained (Positive NTCs/Total NTCs) that were chosen to either show cases (i) where only a small number of NTCs is carried out, even without obtaining positive amplification (from 0/2 to 0/100), or (ii) that were observed in the present study (from 0/279 to 151/1170), or (iii) that were chosen to present hypothetical situations where a very high number of NTCs was performed allowing detection of a few contaminated amplifications (from 50/1000 to 5/1000). For each of these NTCs values, we determined the threshold values of the minimal number of successful sample replications that must be obtained following a maximal number of PCR attempts (Positive Sample PCRs/Total Sample PCRs) to ensure that the chance that all sample amplifications are due to contamination is no more than 5%. The P-value will be lower than 0.05 when more replications are successful or when successful replications are obtained with fewer PCR attempts. When the rate of PCR success required is very high, this minimal number could prevent the validation of sequences obtained from samples that do not show very good DNA preservation and do not yield a product with every PCR attempt. Thus, the table also indicates for these cases the alternative replication number just above this minimal number, which [file pone.0013042.s008.doc]

Table S2: Examples of significantly different distributions between sample and control PCRs

|  | Positive Sample PCRs/Total Sample PCRs | | | | | |
| --- | --- | --- | --- | --- | --- | --- |
| Positive NTCs/Total NTCs | 1 Sample | | 10 Samples Bonferroni | | 50 Samples Bonferroni | |
| 0/2 | 5/5 |  | 19/19 |  | 44/44 |  |
| 0/5 | 2/2 | 3/4 | 6/6 |  | 8/8 |  |
| 0/10 | 2/3 | 3/6 | 3/3 | 4/5 | 4/4 |  |
| 0/20 | 2/6 |  | 2/2 | 3/5 | 3/3 |  |
| 0/50 | 2/15 |  | 2/4 | 3/11 | 2/2 |  |
| 0/100 | 2/29 |  | 2/8 |  | 2/3 | 3/12 |
| 0/279 | 2/81 |  | 2/21 |  | 2/9 |  |
| 0/409 | 2/118 |  | 2/31 |  | 2/14 |  |
| 0/1500 | 2/435 |  | 2/114 |  | 2/49 |  |
| 15/372 | 2/8 |  | 2/2 | 3/8 | 3/5 | 4/10 |
| 151/1170 | 2/3 | 3/6 | 3/3 |  | 4/4 |  |
| 50/1000 | 2/7 |  | 2/2 |  | 4/4 |  |
| 15/1000 | 2/22 |  | 2/6 |  | 2/3 | 3/12 |
| 5/1000 | 2/57 |  | 2/16 |  | 2/7 |  |

When sample and contaminating sequences are indistinguishable, to ensure authenticity of the sample amplification with a 95% confidence level it is necessary to demonstrate that the rate of success of sample amplification is significantly higher than the background of contamination detected in the PCR blank controls (NTCs). The table presents various examples of threshold values that are different with a P-level of significance (α) of 0.05 or lower as determined using the Fisher’s exact test. The left column with the blue background contains examples of various total numbers of NTCs performed and numbers of contaminated PCRs obtained (Positive NTCs/Total NTCs) that were chosen to either show cases (i) where only a small number of NTCs is carried out, even without obtaining positive amplification (from 0/2 to 0/100), or (ii) that were observed in the present study (from 0/279 to 151/1170), or (iii) that were chosen to present hypothetical situations where a very high number of NTCs was performed allowing detection of a few contaminated amplifications (from 50/1000 to 5/1000). For each of these NTCs values, we determined the threshold values of the minimal number of successful sample replications that must be obtained following a maximal number of PCR attempts (Positive Sample PCRs/Total Sample PCRs) to ensure that the chance that all sample amplifications are due to contamination is no more than 5%. The P-value will be lower than 0.05 when more replications are successful or when successful replications are obtained with fewer PCR attempts. When the rate of PCR success required is very high, this minimal number could prevent the validation of sequences obtained from samples that do not show very good DNA preservation and do not yield a product with every PCR attempt. Thus, the table also indicates for these cases the alternative replication number just above this minimal number, which would allow validation of sequences obtained from such less well-preserved samples. Finally, when several samples are compared to a single series of NTCs, there is an increased probability that an apparently significant difference can be obtained by chance and it is necessary to correct for multiple comparisons. This correction was performed using the conservative Bonferroni correction where the P-value α  is adjusted by the number n of samples analyzed using α/n [11]. The table presents the numbers required to validate any sample using three examples of multiple comparisons: either when analyzing a single sample (no correction), or 10 or 50 samples.
